# Qualitative and quantitative educational disparities and brain signatures in healthy aging and dementia across global settings

**DOI:** 10.1016/j.eclinm.2025.103187

**Published:** 2025-04-10

**Authors:** Raul Gonzalez-Gomez, Josephine Cruzat, Hernán Hernández, Joaquín Migeot, Agustina Legaz, Hernando Santamaria-García, Sol Fittipaldi, Marcelo Adrián Maito, Vicente Medel, Enzo Tagliazucchi, Pablo Barttfeld, Daniel Franco-O’Byrne, Ana María Castro Laguardia, Patricio A. Borquez, José Alberto Avila-Funes, María I. Behrens, Nilton Custodio, Temitope Farombi, Adolfo M. García, Indira Garcia-Cordero, Maria E. Godoy, Cecilia Gonzalez Campo, Kun Hu, Brian Lawlor, Diana L. Matallana, Bruce Miller, Maira Okada de Oliveira, Stefanie D. Pina-Escudero, Elisa de Paula França Resende, Pablo Reyes, Andrea Slachevsky, Leonel T. Takada, Görsev G. Yener, Carlos Coronel-Oliveros, Agustin Ibañez

**Affiliations:** a Latin American Brain Health Institute (BrainLat), Universidad Adolfo Ibañez, Santiago de Chile, Chile; b Global Brain Health Institute (GBHI), University of California, San Francisco, San Francisco, CA, USA; c Cognitive Neuroscience Center, Universidad de San Andrés, Buenos Aires, Argentina; d Pontificia Universidad Javeriana, Bogotá D.C., Colombia; e Hospital Universitario San Ignacio, Center for Memory and Cognition, Intellectus, Bogotá D.C., Colombia; f School of Psychology, Trinity College Dublin, Ireland; g Departamento de Física, Universidad de Buenos Aires, Argentina; h Instituto de Investigaciones Psicológicas, Córdoba, Argentina; i Colegio Suizo de Santiago, Santiago, Chile; j Dirección de Enseñanza, Instituto Nacional de Ciencias Médicas y Nutrición, Salvador Zubirán, Ciudad de México, México; k Faculty of Medicine, University of Chile, Santiago, Chile; l Centro de Investigación Clínica Avanzada (CICA), Universidad de Chile, Santiago, Chile; m Servicio de Neurología, Departamento de Medicina, Clínica Alemana-Universidad del Desarrollo, Santiago de Chile, Chile; n Departamento de Neurología y Neurocirugía, Hospital Clínico Universidad de Chile, Santiago, Chile; o Unit Cognitive Impairment and Dementia Prevention, Peruvian Institute of Neurosciences, Lima, Peru; p Departamento de Lingüística y Literatura, Universidad de Santiago de Chile, Santiago, Chile; q Tanz Centre for Research in Neurodegenerative Diseases, University of Toronto, Toronto, Canada; r Consejo Nacional de Investigaciones Científicas y Técnicas (CONICET), Buenos Aires, Argentina; s Department of Anesthesia, Critical Care and Pain Medicine, Massachusetts General Hospital, Harvard Medical School, Boston, MA, USA; t Trinity College Institute of Neuroscience (TCIN), Trinity College Dublin, Dublin, Ireland; u Instituto de Envejecimiento, Facultad de Medicina, Pontificia Universidad Javeriana, Bogotá D.C., Colombia; v Center for Memory and Cognition, Hospital Universitario San Ignacio Bogotá, San Ignacio, Bogotá D.C., Colombia; w Departamento de Salud Mental, Hospital Universitario Fundación Santa Fe de Bogotá, Bogotá D.C., Colombia; x Memory and Aging Center, Department of Neurology, University of California, San Francisco, CA, USA; y Cognitive Neurology and Behavioral Unit (GNCC), University of São Paulo, São Paulo, Brazil; z Universidade Federal de Minas Gerais, Belo Horizonte, Minas Gerais, Brazil; aa Geroscience Center for Brain Health and Metabolism (GERO), Santiago de Chile, Chile; ab Memory and Neuropsychiatric Center (CMYN), Neurology Department, Hospital del Salvador, Santiago de Chile, Chile; ac Neuropsychology and Clinical Neuroscience Laboratory (LANNEC), Physiopathology Program – Institute of Biomedical Sciences (ICBM), University of Chile, Santiago, Chile; ad Universidade de São Paulo, São Paulo, Brazil; ae Faculty of Medicine, Dokuz Eylul University, Izmir, Turkey; af Brain Dynamics Multidisciplinary Research Center, Dokuz Eylul University, Izmir, Turkey; ag Izmir Biomedicine and Genome Center, Izmir, Turkey; ah Trinity College Dublin, Dublin, Ireland

**Keywords:** Educational disparities, Education quality, Years of education, Brain health, Aging and dementia

## Abstract

**Background:**

While education is crucial for brain health, evidence mainly relies on individual measures of years of education (YoE), neglecting education quality (EQ). The effect of YoE and EQ on aging and dementia has not been compared.

**Methods:**

We conducted a cross-sectional assessment of the effect of EQ and YoE on brain health in 7533 subjects from 20 countries, including healthy controls (HCs), Alzheimer's disease (AD), and frontotemporal lobar degeneration (FTLD). EQ was based on country-level quality indicators provided by the programme for international student assessment (PISA). After applying neuroimage harmonization, we examined its effect, along with YoE, on gray matter volume and functional connectivity. Regression models were adjusted for age, sex, and cognition, controlling for multiple comparisons. The influence of image quality was assessed through sensitivity analysis. Data collection was conducted between June 1 and October 30, 2024.

**Findings:**

Less EQ and YoE were associated with brain alterations across groups. However, EQ had a stronger influence, mainly targeting the critical areas of each condition. At the whole-brain level, EQ influenced volume (HCs: Δmean = 2·0 [1·9–2·0] × 10^−2^, *p* < 10^−5^; AD: Δmean = 0·1 [−0·0 to 0·3] × 10^−2^, *p* = 0·18; FTLD: Δmean = 3·5 [3·0–4·0] × 10^−2^, *p* < 10^−5^; all with 95% confidence intervals) and networks (HCs: Δmean = 13·5 [13·2–13·7] × 10^−2^, *p* < 10^−5^; AD: Δmean = 5·9 [5·2–6·7] × 10^−2^, *p* < 10^−5^; FTLD: Δmean = 13·2 [11·2–13·7] × 10^−2^, *p* < 10^−5^) 1·3 to 7·0 times more than YoE. These effects remain robust despite variations in income and socioeconomic factors at country and individual levels.

**Interpretation:**

The results support the need to incorporate education quality into studying and improving brain health, underscoring the importance of country-level measures.

**Funding:**

Multi-partner consortium to expand dementia research in Latin America (ReDLat).

**Research in context**

**Evidence before this study**

Education has been linked to substantial brain changes; however, the methods used to measure its effects remain inefficient. A search of academic databases (e.g., PubMed, Scopus, Web of Science) revealed that neuroscience typically operationalizes education as years of schooling, overlooking education quality. Despite decades of research, the influence of this dimension on the brain still needs to be better understood. Although access to this individual-level education quality is limited, measures are available at the country level. This offers an opportunity to compare the effects of educational disparities on brain health at individual and country scales.

**Added value of this study**

We analyzed a large sample of neuroimaging data from 7533 subjects across 20 countries, providing evidence of the effect of education on brain structure and function. Additionally, we examined how this influence varies across aging and dementia phenotypes, comparing two dimensions of education: quality and quantity. Education quality was assessed using country-standardized quality indicators. Our findings show that quality influences gray matter volume and functional networks 1·3 to 7 times more than quantity.

**Implications of all the available evidence**

Regarding brain health, the present findings suggest that fewer years of schooling in a country with a high-quality education could be more beneficial than completing more years in a low-quality education. This evidence could be helpful for future research and policymakers, highlighting the benefits of enhancing education quality for future generations.

## Introduction

Education is a cornerstone of socioeconomic status and cognitive reserve, playing a critical role in brain health, particularly in aging and the risk/prevalence of dementia.^1^, ^2^, ^3^ Evidence shows that educational inequalities are biologically embedded and manifest as reduced brain volume and connectivity.^3^ These effects are amplified in countries with higher socioeconomic disparities.^3^ However, most research on brain health relies on quantitative assessments measured in years of education (YoE). This approach disregards the influence of education quality (EQ), undermining the validity of comparisons across countries with diverse educational systems. For instance, one year in the United States of America may equate to three years in low-income countries,^4^ making cross-country comparisons of YoE deficient. Despite growing awareness of the role of education in brain health, the specific contribution of EQ has not been explored, especially in the context of aging and dementia.

A relatively new research agenda^5^ focusing on aggregate-level measures (country, city, or neighborhood) rather than individual metrics provides evidence of how macro-level inequalities shape brain health signatures.^6^, ^7^, ^8^, ^9^ Although some studies have compared these measures to individual-level inequalities,^6^^,^^7^ none has been developed concerning educational disparities.^6^, ^7^, ^8^, ^9^ Specifically, it remains unclear whether qualitative educational disparities affect brain health differently. The Programme for international student assessment (PISA) is the only available qualitative measure explicitly designed for cross-national comparability of EQ.^10^^,^^11^ The United Nations's sustainable development goals identify learning outcomes in reading and mathematics as key indicators of education quality—areas measured by PISA, along with science (https://www.oecd.org/pisa). International institutions such as the United Nations, UNESCO, the World Bank, the International Monetary Fund, the Organization for Economic Co-operation and Development, and the Inter-American Development Bank rely on PISA as a benchmark.^10^^,^^11^ No other globally validated assessment of education quality exists. This index was designed to capture the diversity of educational systems, ensuring representative sampling within each country.^10^^,^^11^ Although not exempt from limitations,^11^ it is a standardized, national-level measure of education quality^10^^,^^11^ and has been shown to correlate with gross domestic product, income, and work productivity.^10^, ^11^, ^12^

Here, we compare the effect of EQ- and YoE-brain associations on aging and dementia in 7533 subjects from 20 countries, encompassing healthy controls (HCs), Alzheimer's disease (AD), and frontotemporal lobar degeneration (FTLD) patients. We examine their effect on gray matter volume and functional connectivity at the regional and whole-brain level using T1-weighted (T1w) and resting-state functional magnetic resonance images (rs-fMRI). We used regression models to compare the associations of these factors with neural correlates, adjusting all analyses for age, sex, and cognition as covariates. The potential variability introduced by different scanners was mitigated using harmonization frameworks. Moreover, we ruled out possible effects of image quality on our results through sensitivity analyses. We expect a general effect of the biological embedding of educational inequalities in brain measures, with more substantial influence of EQ than YoE, also revealing distinct patterns on aging and dementia phenotypes across global contexts.

## Methods

### Study design

Following previous works,^3^^,^^6^^,^^7^^,^^9^ this study uses a cross-sectional observational analysis using multicentric neuroimages. Each dataset originated from independent research initiatives that applied random or standardized selection methods to ensure representativeness. The sampling procedures are detailed in the original studies, referenced in Supplementary Materials S1. The large number of participants improves the stability of estimates and enhances generalizability across diverse populations. However, our samples are clinical and are not intended to be representative of the general population.^6^^,^^7^^,^^9^ After merging the datasets, participants were selected based on age, diagnosis (HCs, AD, or FTLD), and education (between 0 and 20 years of schooling). Exclusion criteria minimize confounding factors like missing data, unrelated neurological conditions, and deficient image quality. In this study, we used recognized and widely utilized research datasets, especially for dementia, such as ADNI and NIFD, which follow standardized protocols and are extensively used in neuroimaging research^3^^,^^7^^,^^9^ to ensure methodological consistency and comparability across studies.

### Participants

**Fig. 1:**
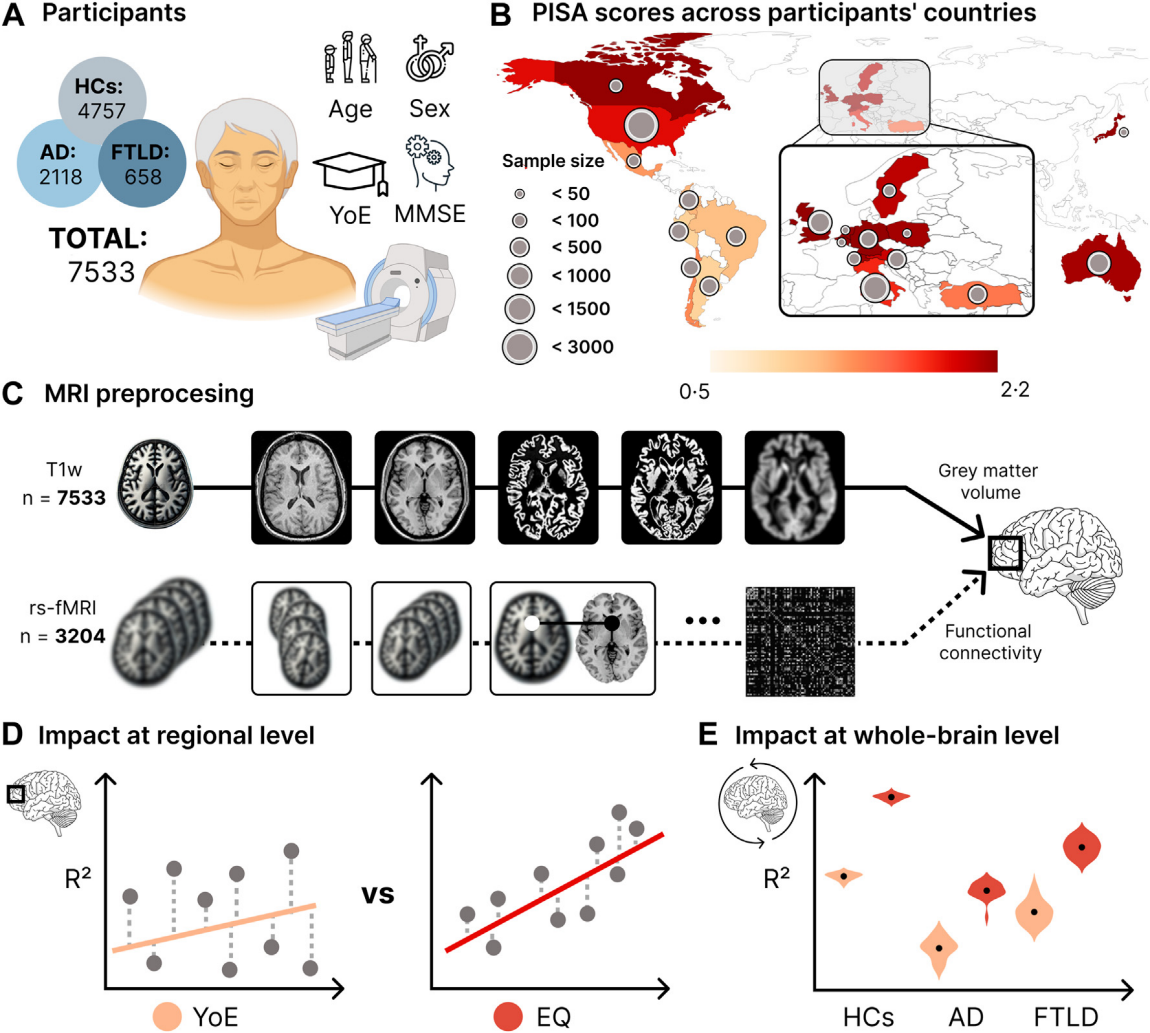
Participants and data analysis pipeline **(A)** The dataset included healthy controls, patients with Alzheimer's disease, and frontotemporal lobar degeneration. T1w scans were available for all participants (n = 7533), and rs-fMRI recordings were acquired for a subsample of 3204 subjects **(B)** Sample size and education quality (measured through PISA scores) for the 20 countries included in the dataset **(C)** Images preprocessing pipeline. The analyses included regional **(D)** and whole-brain **(E)** measures. Local measures consist of voxel gray matter and pairwise functional connectivity correlations. Whole-brain measures are the average gray matter volume and the global efficiency of the functional networks. All analyses were conducted separately for each group and corrected by multiple comparisons. *Abbreviations*: AD: Alzheimer's disease, EQ: education quality, FTLD: frontotemporal lobar degeneration, HCs: healthy controls, PISA: Programme for International Student Assessment, rs-fMRI: resting-state functional magnetic resonance images, T1w: T1-weighted, YoE: years of education.

A total of 7533 participants from 20 countries were included: Argentina, Australia, Austria, Belgium, Brazil, Canada, Chile, Colombia, Germany, Italy, Japan, Mexico, Netherlands, Peru, Poland, Sweden, Switzerland, Turkey, United Kingdom, and the United States of America. Supplementary Material S1 provides details on the data's origin and sample size. Participants were categorized into three groups based on predefined clinical criteria—HCs (n = 4757), AD (n = 2118), and FTLD (n = 658)—to study the effects of educational disparities on aging, as well as the most common causes of late-onset and early-onset dementia, respectively.^13^^,^^14^ HCs had no history of psychiatric or neurological conditions and patients with AD^13^ and FTLD^14^ received their diagnosis following internationally recognized diagnostic guidelines for these dementias, ensuring consistency in classification. FTLD includes the following clinical subtypes: behavioral variant frontotemporal dementia, semantic variant primary progressive aphasia, nonfluent/agrammatic variant primary progressive aphasia, corticobasal syndrome, and progressive supranuclear palsy. These dementia's typical grey matter volume reduction patterns were confirmed using voxel-based morphometry (Supplementary Material S2). Cognitive status was assessed using the Mini-Mental State Examination (MMSE). Fig. 1 presents the included samples, the PISA scores of the countries within the sample, and the data analysis pipeline. Furthermore, Table 1 details the participants' demography and cognitive assessments for samples with T1w and fMRI. The local institutions approved the data acquisition protocols, and all participants provided informed consent per the Declaration of Helsinki.

**Table 1:** Demographic and cognitive characteristics of subjects in T1w and rs-fMRI samples.

Group	n	Age			Sex	YoE			MMSE		
		Mean	SD	Range	Females %	Mean	SD	Range	Mean	SD	Range
	T1w										
HCs	4757	58·1	17·6	20–100	56·6	13·7	4·6	0–20	29·1	1·7	20–30
AD	2118	72·2	7·9	40–98	59·0	11·5	4·6	0–20	23·2	5·3	0–30
FTLD	658	65·7	8·2	40–87	46·4	13·8	4·6	0–20	22·6	5·8	2–30
	rs-fMRI										
HCs	1863	63·9	13·5	21–100	58·3	15·8	2·9	1–20	28·9	1·3	21–30
AD	587	70·8	8·5	40–98	60·6	13·0	4·9	1–20	21·1	5·0	2–30
FTLD	563	64·8	8·1	40–87	46·1	14·2	4·4	0–20	22·7	5·4	3–30

*Abbreviations*: MMSE: Mini-Mental State Examination, SD: standard deviations, YoE: years of education.

### Education metrics

EQ was quantified using country-level scores from the PISA, a standardized evaluation of competencies in reading, mathematics, and science. This test is administered to a representative sample of 15-year-old students in countries that are members or associates of the Organization for Economic Cooperation and Development (OECD).^10^^,^^11^ The PISA sampling methodology is designed to capture the diversity within each country's educational system, ensuring that the results reflect national averages and within-country variations in education quality.^10^^,^^11^ This approach allows PISA to account for country disparities in school performance while maintaining a globally standardized measure of education quality (https://www.oecd.org/pisa).^10^^,^^11^ Results are reported on a standardized scale with a mean of 500 and a standard deviation of 100.^10^^,^^11^ Assessments were conducted in 2003, 2006, 2009, 2012, 2015, 2018, and 2022, ensuring comparability across cycles and countries through rigorous item response theory.^10^^,^^11^ The assessment is adaptive, meaning that question difficulty is adjusted to ensure that a score of 500 in one country reflects the same level of competency as a score of 500 in another. Additionally, PISA employs extensive validation processes, including linking items across test cycles and equating methodologies, to maintain score consistency and cross-national equivalence.^10^^,^^11^ Additional details and open access to the data are available on the official project website (https://www.oecd.org/pisa). Among the countries entered in this study, most reported data for all assessment cycles, except for Argentina, Colombia, and Peru, which have data for only 4, 6, and 5 cycles, respectively. To compute EQ metrics, PISA results for these countries were averaged annually. Scores were standardized to z-scores (mean = 0, standard deviation = 1) by subtracting 500 and dividing by 100. A constant of 2 was added to eliminate negative values, as 95·4% of the data in the z-distribution range between −2 and 2. We tested the temporal stability of PISA using a mixed linear model. This model included an interaction term (year x country) and a random effect by country (1|country) to predict PISA scores. Results showed no significant changes in PISA scores over time, confirming the stability of EQ across years (Supplementary Material S3). Each country's EQ value was calculated as the average of these scores across assessment years. The resulting values (Fig. 1B) have a mean of 1·83, a standard deviation of 0·51, and range from 0·93 to 2·61. For comparison, YoE was measured as the total number of years of formal schooling completed by each participant at any institution.

### MRI acquisition, preprocessing, and calculation of brain metrics

T1w images were acquired for all subjects, while rs-fMRI recordings were obtained for a subset of 3204 participants, comprising 2056 HCs, 585 AD, and 563 FTLD. We assessed the data quality for all images by careful visual inspection and computational algorithms. MRIQC software^15^ was used to compute no-reference image quality metrics, including the signal-to-noise ratio (SNR) for T1w and rs-fMRI, as well as the temporal signal-to-noise ratio (tSNR) for rs-fMRI. These metrics were used in the sensibility analysis to discard any possible side effects due to image quality. The official website provides details on the software and instructions on how to compute these metrics (https://mriqc.readthedocs.io). T1w and rs-fMRI were recorded for the whole brain with variable recording protocols. However, images were normalized during preprocessing through a robust two-step intrasubject harmonization process.^3^

T1w images were preprocessed and analyzed using the voxel-based morphometry method with the Computational Anatomy (CAT12) toolbox (https://neuro-jena.github.io/cat) in MATLAB R2022a. The preprocessing pipeline included bias-field correction, noise reduction, skull stripping, segmentation, and normalization to the Montreal Neurological Institute (MNI) space at a 1·5 mm isotropic resolution and modulation, all following the default parameters of the toolbox. CAT12 normalizes the grey matter segmentations to the mean global intensity of each subject to achieve intra-subject harmonization.^3^ Subsequently, these segmentations were harmonized again using a min–max scaling to a range of 0–1. This pipeline provides regional brain measures for voxel-wise gray matter volume standardized to the same number of voxels for each subject. We then calculated a whole-brain measure to evaluate the overall gray matter integrity by averaging gray matter volume across all voxels for each subject. Analyses on grey matter volume, following common neuroimaging practices,^3^ were adjusted for total intracranial volume (TIV). This adjustment ensures that head size does not influence our results.

The rs-fMRI data was processed using the fmriprep (version 22·0·2) standardized pipeline to maximize reproducibility and accuracy.^16^ This pipeline includes brain extraction, head motion correction, slice timing correction, coregistration, normalization, field distortion correction, and confound estimation. Further details are available on the official website (https://fmriprep.org). Subsequently, white matter and cerebrospinal fluid confounds were removed, and a band-pass filter between 0·01 and 0·1 Hz was applied to reduce noise.^17^ Time series for each region of interest (ROI) in the Automated Anatomical Labeling (AAL) atlas^18^ were then extracted using the Nilearn library (v0·10·0) in Python v3·8. Time series were adjusted to 120 volumes and normalized using the z-score method, which scales the data with a mean of 0 and a standard deviation of 1.^17^ The harmonized time series were used to compute functional connectivity matrices using Pearson correlation.^3^^,^^17^ In this case, brain measures at regional levels were the Pearson correlation coefficient between each possible pair of ROI. These coefficients, ranging from −1 to 1, serve as the second step of intra-subject harmonization. Furthermore, we utilized global efficiency metrics from graph theory^17^^,^^19^ to compute a measure that characterizes the whole-brain network.^17^^,^^19^ This metric has been widely used in studying aging and dementia,^20^ as it captures effective information transmission throughout the brain.^17^^,^^19^ Following standard methods of network analyses,^19^ we binarized the functional connectivity matrices using a proportional threshold of 0·3. Then, we calculated global efficiency using the Brain Connectivity Toolbox for Python (v0·6·1) as the inverse of the average shortest path length across all nodes in the network.^19^ In graph theory, each node represents an ROI in the AAL atlas, and the shortest path length is the minimum number of connections needed to travel from one node to another.^19^ Analyses on functional connectivity were adjusted for resting-state recording conditions (eyes open or closed). This adjustment minimizes variability related to the recording state and ensures that observed effects are not driven by differences in participants' vigilance or arousal levels.^3^

### Statistical analysis

All analyses were conducted separately for each group, as well as for gray matter and functional variables. First, we applied multiple ordinary least squares regressions at the regional level to predict brain measures using either EQ or YoE. Model performance was evaluated using adjusted R^2^, with p-values corrected for multiple comparisons using the False Discovery Rate (FDR) threshold set at 0·05.^3^ The adjusted R^2^ balances the sample size and the number of predictors. It is defined as $R_{adj}^{2}=1-\left(\frac{\left(1-R^{2}\right)\left(n-1\right)}{n-p-1}\right)$, where *n* represents the sample size and *p* is the number of predictors. Only voxels or connections with an R^2^ value greater than 0·05 were analyzed. We also checked the normality of the data using the Shapiro–Wilk test (*p* > 0·11) and the homoscedasticity through the Breusch–Pagan test (*p* > 0·23). Using Wilcoxon signed-rank tests for repeated measures, we compared the adjusted R^2^ from the EQ and YoE effects to assess their magnitude. Additionally, we used Cohen's d effect size, which is more stable for large sample sizes. Second, ridge regression was employed at the whole-brain level to predict whole-brain measures based on either EQ or YoE. This approach ensures regularization to prevent overfitting, providing robust estimates of the relationships between educational factors and brain metrics while being resilient to multicollinearity.^21^ Model performance was assessed by the R^2^ regression between the predicted and observed whole-brain brain measures using data partition (3-fold cross-validation) up to 20 repetitions.^21^ We included hyperparameter tuning by sweeping 1000 alpha values from 10^−10^ to 10^10^ logarithmically spaced using grid search. The mean R^2^ across folds was calculated and stored for each iteration. These results from 20 repetitions were compared for EQ and YoE across groups. We used a combination of permutation tests and bootstrapping, up to 10^5^ iterations, to assess the statistical significance of the differences between factors and to calculate 95% confidence intervals. To quantify the change in magnitude, we used Δmean, calculated as the average difference between the R^2^ values of both effects across all permutations. All analyses controlled for age, sex, and cognition (measured by MMSE) to account for aging, gender differences, and cognitive function. Each modality was also controlled for nuisance factors: TIV for grey matter volume and resting-state conditions for functional connectivity. We replicated whole-brain analyses to compare EQ with its interaction with YoE, calculated as the product of both variables (EQ × YoE). Additionally, we assessed potential image quality effects by correlating the SNR and tSNR values with EQ and YoE. Correlations with r coefficients ranging from −0·1 to 0·1 were considered negligible. Also, we corrected whole-brain measures for image quality metrics using a general linear model and repeated the corresponding analyses with these adjusted values.

### Role of the funding source

The data collection for subjects from Latin America was funded by the ReDLat consortium; however, ReDLat had no role in the writing of the manuscript or the decision to submit it for publication.

## Results

### Qualitative and quantitative education differentially affect brain burden across groups

**Fig. 2:**
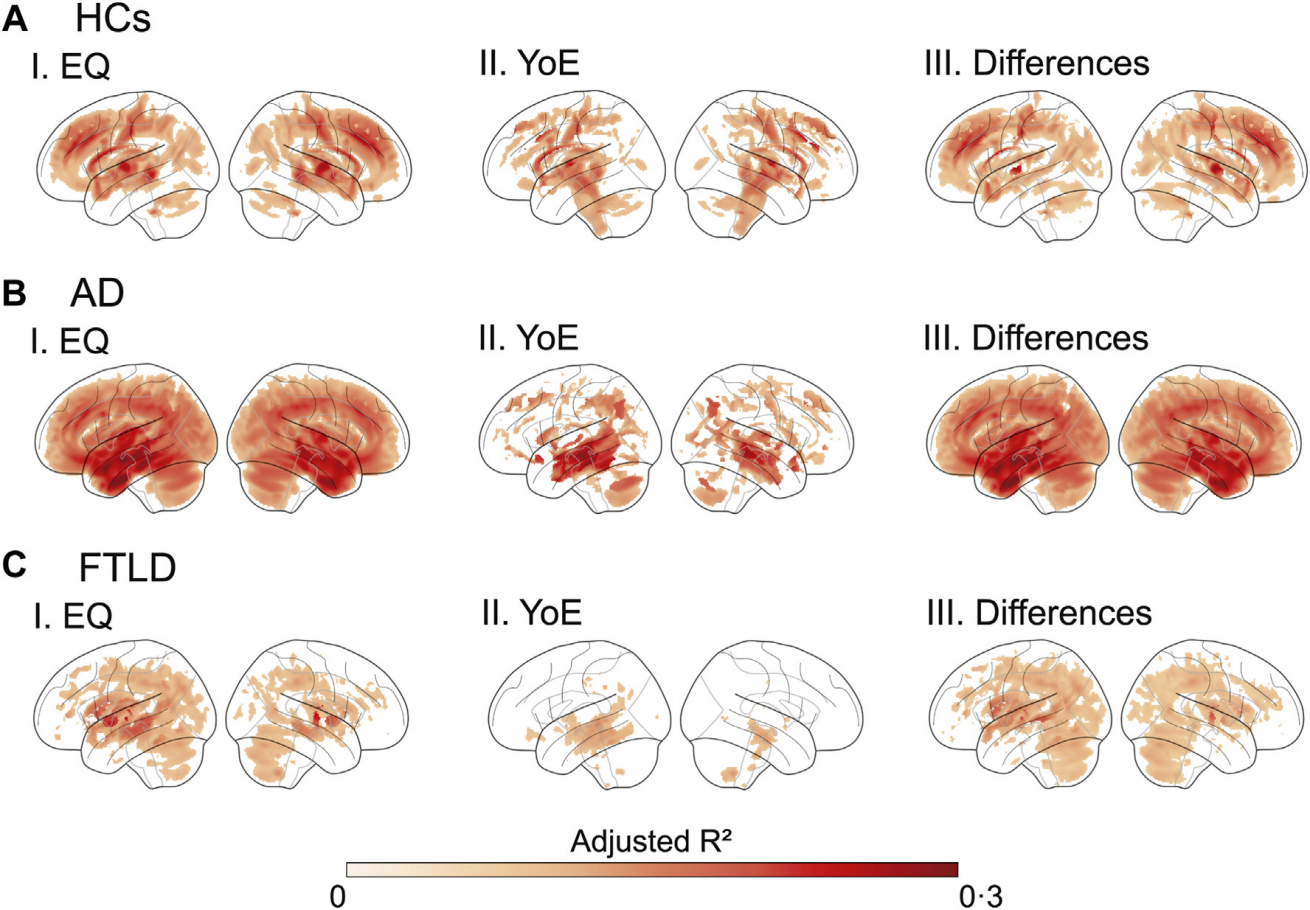
Effect of education on voxel-wise gray matter volume. Analyses were conducted separately for **(A)** healthy controls, **(B)** Alzheimer's disease, and **(C)** frontotemporal lobar degeneration groups. Voxel-wise gray matter volume was predicted using ordinary least squares regression based on (I) Education Quality (EQ) or (II) Years of Education (YoE), with age, sex, cognition and total intracranial volume (TIV) as covariates. Multiple corrections were applied using the False Discovery Rate (p_FDR_ < 0·05). Additionally, panel III presents the adjusted R^2^ difference between EQ and YoE predictions (I–II). *Abbreviations*: AD: Alzheimer's disease, EQ: education quality, FTLD: frontotemporal lobar degeneration, HCs: healthy controls, YoE: years of education.

Less EQ and YoE were associated with reduced brain volume and functional connectivity across groups; however, EQ demonstrated a more substantial and widespread effect (Supplementary Materials S4 and S5). Grey matter volume analysis analyses at the regional level showed a considerable increase in the number of significant voxels for EQ than YoE (Supplementary Table S6), as well as higher R^2^ values), as well as higher adjusted R^2^ values (HCs: *W* = 1·5 × 10^10^, *p* < 10^−6^; Cohen's d = 1·2; AD: *W* = 2·9 × 10^10^, *p* < 10^−6^, Cohen's d = 0·8; FTLD: *W* = 7·3 × 10^9^, *p* < 10^−6^, Cohen's d = 0·64). Fig. 2 displays the effect maps for each predictor and their differences across groups, with detailed statistical values provided in Supplementary Materials S4. While YoE primarily affected the temporal lobes and cerebellum across all groups (Fig. 2, subpanels I), EQ extended its influence on additional regions (Fig. 2, subpanels II and III). In HCs, EQ had a stronger influence on the parietal, occipital, and frontal areas (Supplementary Materials S4). Its effects were more extensive in AD, particularly in the temporal lobe (Supplementary Materials S4). In contrast, in FTLD, EQ predominantly influenced the frontal, parietal, and occipital regions, with temporal lobe involvement (Supplementary Materials S4).

**Fig. 3:**
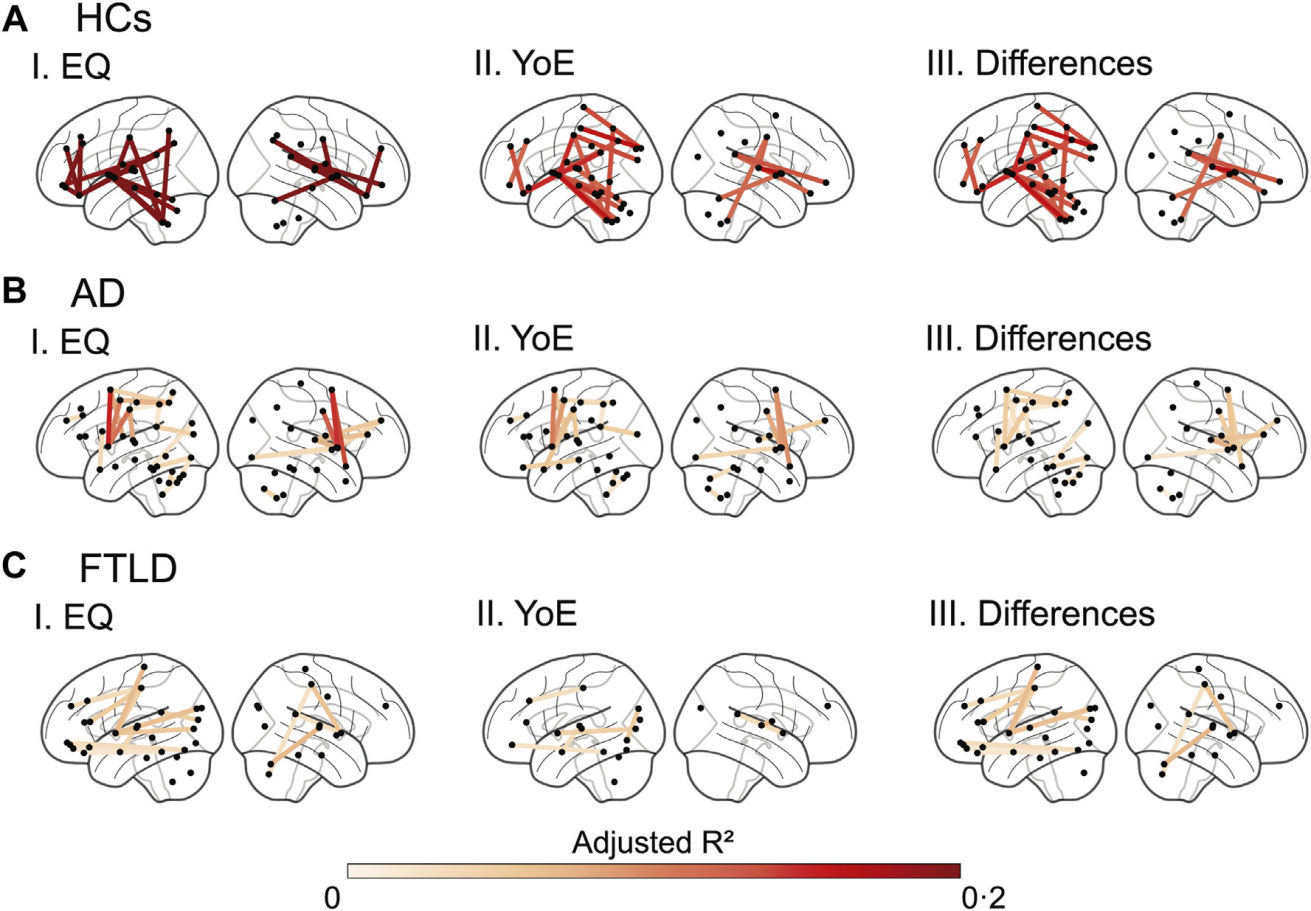
Effect of education on pairwise functional connectivity. Analyses were conducted separately for **(A)** healthy controls, **(B)** Alzheimer's disease, and **(C)** frontotemporal lobar degeneration groups. Pairwise functional connectivity was predicted using ordinary least squares regression based on (I) Education Quality (EQ) or (II) Years of Education (YoE), with age, sex, cognition, and resting-state recording conditions (eyes open or closed) as covariates. Multiple corrections were applied using the False Discovery Rate (p_FDR_ < 0·05). Additionally, panel III presents the adjusted R^2^ difference between EQ and YoE predictions (I–II). Only connections exceeding 70% of the maximum connection strength are displayed. *Abbreviations*: AD: Alzheimer's disease, EQ: education quality, FTLD: frontotemporal lobar degeneration, HCs: healthy controls, YoE: years of education.

Network analysis also highlighted significant results for both predictors (Supplementary Material S5); however, EQ affected more numbers of connections (Supplementary Table S7) and exhibited higher adjusted R^2^ values (HCs: *W* = 5·2 × 10^6^, *p* < 10^−3^, Cohen's d = 1·3; AD: *W* = 2·6 × 10^4^, *p* < 10^−3^, Cohen's d = 0·8; FTLD: *W* = 1·3 × 10^4^, *p* < 10^−3^, Cohen's d = 0·4). Fig. 3 displays the connectivity maps for each predictor and their differences across groups, with detailed statistical values provided in Supplementary Materials S5. YoE was primarily associated with changes in connectivity involving temporal and cerebellar networks (Fig. 3, subpanels I), whereas EQ extended these effects to additional regions (Fig. 2, subpanels II and III). Among HCs, EQ strongly influenced the cerebellar network and fronto-parietal connections (Supplementary Material S5). In AD, EQ's effects were most pronounced in intra-frontal and posterior networks (Supplementary Material S5). For patients with FTLD, orbitofrontal-temporal and intra-temporal connections were the most affected (Supplementary Material S5).

### Quantifying the effect of education quality and quantity

**Fig. 4:**
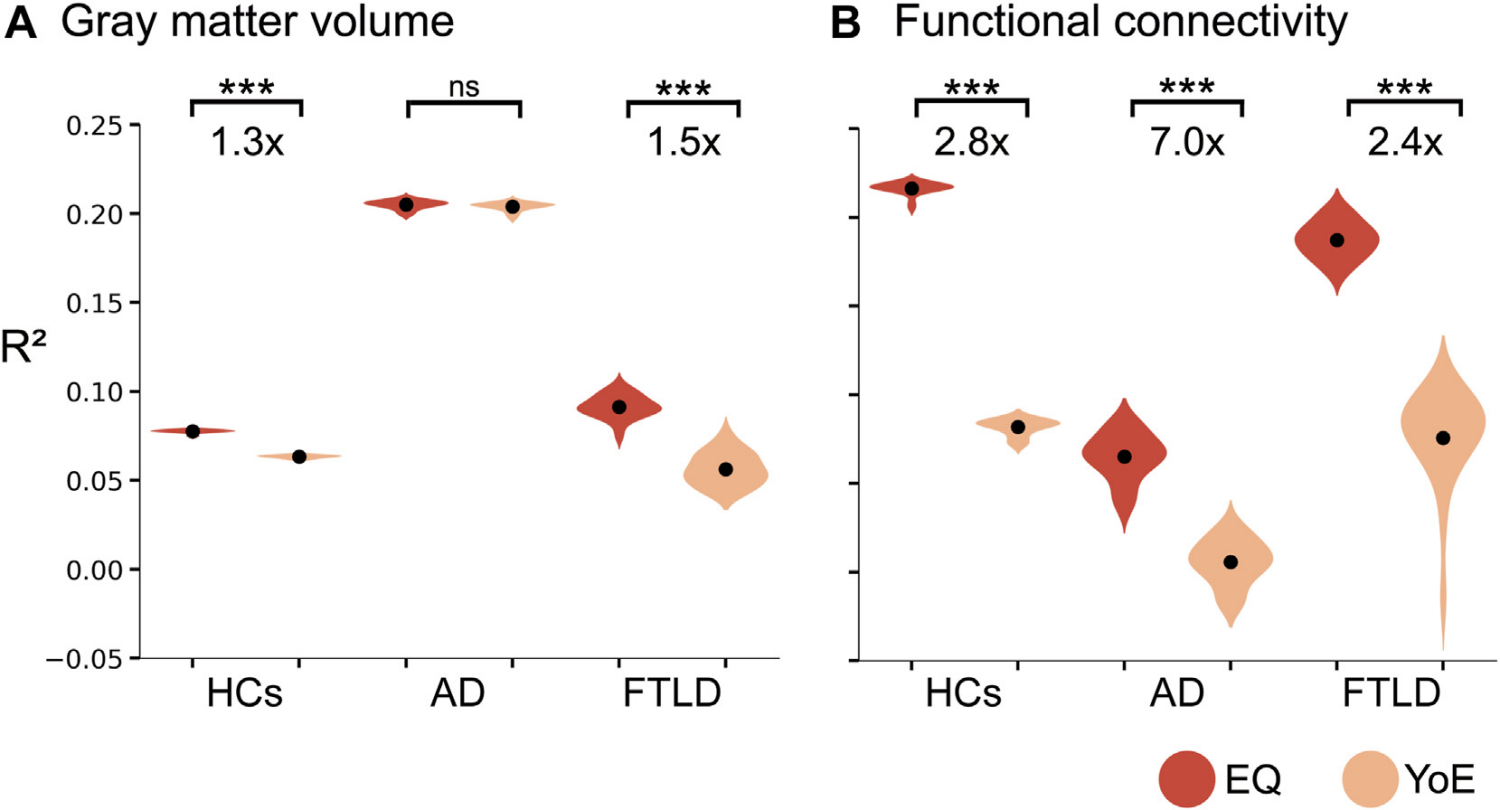
Effect of education on whole-brain measures of (**A**) grey matter volume and (**B**) global efficiency of networks. Analyses were conducted separately for healthy controls, Alzheimer's disease, and frontotemporal lobar degeneration groups. The average gray matter volume and global efficiency of the functional network were predicted from Education Quality (EQ) or years of education (YoE) using ridge regression with data partition (3-fold cross-validation up to 20 repetitions). Adjustments were made for age, sex, and cognition. Grey matter integrity and functional connectivity analysis were also adjusted for total intracranial volume (TIV) and resting-state recording conditions (open or closed eyes), respectively. Corrections were applied using the False Discovery Rate (*p*_FDR_ < 0·05). *Abbreviations*: ∗∗∗: *p* < 0·001, AD: Alzheimer's Disease, EQ: education quality, FTLD: frontotemporal lobar degeneration, HCs: healthy controls, ns: not significant, YoE: years of education.

At the whole-brain level, EQ demonstrated a more substantial influence across groups and modalities (Fig. 4). Grey matter volume analysis analyses showed a higher effect of EQ than YoE for HCs (Δmean = 2·0 [1·9–2·0] × 10^−2^, *p* < 10^−5^, with 95% confidence intervals) and FTLD (Δmean = 3·5 [3·0–4·0] × 10^−2^, *p* < 10^−5^). These effects were quantified as the ratio mean R^2^_EQ_/mean R^2^_YoE_, showing that EQ's effect is 1·3 and 1·5 times greater in HCs and FTLD, respectively (Fig. 4). In contrast, no significant differences were observed for AD (Δmean = 0·1 [−0·0 to 0·3] × 10^−2^, *p* = 0·18). Network analyses revealed statistical differences in all groups (HCs: Δmean = 13·5 [13·2–13·7] × 10^−2^, *p* < 10^−5^; AD: Δmean = 5·9 [5·2–6·7] × 10^−2^, *p* < 10^−5^; FTLD: Δmean = 13·2 [11·2–13·7] × 10^−2^, *p* < 10^−5^), with EQ having 2·8, 7·0, and 2·4 times greater effect in HCs, AD, and FTLD, respectively.

### Sensitivity analyses

We performed different sensitivity analyses to confirm the robustness of our results. First, we compared the effect of EQ and the interaction (EQ × YoE). In the grey matter volume analysis, EQ surpasses the effect of the interaction (Supplementary Material S8), neither in terms of the number of significant voxels (Supplementary Material S6) or adjusted R^2^ values (HCs: *W* = 2·3 × 10^6^, *p* < 10^−3^, Cohen's d = 0·5; AD: *W* = 1·1 × 10^4^, *p* < 10^−3^, Cohen's d = 0·4; FTLD: *W* = 0·3 × 10^4^, *p* < 10^−3^, Cohen's d = 0·3). This pattern was replicated in the functional analysis, where EQ also exceeded the effect of the interaction (Supplementary Material S9) in terms of the number of significant connections (Supplementary Material S7) or adjusted R^2^ values (HCs: *W* = 6·4 × 10^3^, *p* < 10^−3^, Cohen's d = 0·4; AD: *W* = 3·4 × 10^2^, *p* < 10^−3^, Cohen's d = 0·4; FTLD: *W* = 2·2 × 10^2^, *p* < 10^−3^, Cohen's d = 0·3). These results were replicated at whole-brain level (Supplementary Material S10) in the volume (HCs: Δmean = 1·4 [1·4–1·5] × 10^−2^, *p* < 10^−5^; AD: Δmean = 0·1 [0–0·2] × 10^−2^, *p* < 10^−5^; FTLD: Δmean = 1·6 [1·2–2·0] × 10^−2^, *p* < 10^−5^, all with 95% confidence intervals) and network analysis (HCs: Δmean = 4·9 [4·7–5·1] × 10^−2^, *p* < 10^−5^; AD: Δmean = 2·0 [1·2–2·8] × 10^−2^, *p* < 10^−5^; FTLD: Δmean = 11·2 [9·4–14·0] × 10^−2^, *p* < 10^−5^).

Second, we replicate the main effects of EQ and YoE in a whole-brain analysis, adjusting for income and socioeconomic values at country and individual levels. EQ remains significantly greater than YoE in HCs and FTLD, even after correcting for Gini and gross domestic product indexes in volume (HCs: Δmean = 4·7 [4·6–4·8] × 10^−2^, *p* < 10^−5^; AD: Δmean = 0·2 [−0·05 to 0·4] × 10^−2^, *p* = 0·14; FTLD: Δmean = 2·6 [2·0–3·3] × 10^−2^, *p* < 10^−5^, all with 95% confidence intervals) and functional connectivity (HCs: Δmean = 2·3 [2·2–2·4] × 10^−2^, *p* < 10^−5^; AD: Δmean = 0·1 [−0·04 to 0·6] × 10^−2^, *p* = 0·67; FTLD: Δmean = 0·3 [0·1–0·7] × 10^−2^, *p* < 10^−2^), see details in Supplementary Material S11. After adjusting for individual socioeconomic status, all main effects were replicated (grey matter; HCs: Δmean = 0·9 [0·3–1·5] × 10^−2^, *p* < 10^−3^; AD: Δmean = 2·8 [−1·5 to 1·7] × 10^−2^, *p* = 0·93; FTLD: Δmean = 2·6 [0·8–5·0] × 10^−2^, *p* < 10^−5^, all with 95% confidence intervals; functional connectivity; HCs: mean = 4·3 [2·6–5·9] × 10^−2^, *p* < 10^−5^; AD: Δmean = 3·4 [2·2–4·5] × 10^−2^, *p* < 10^−5^; FTLD, Δmean = 3·3 [0·4–0·6] × 10^−2^, *p* < 0·05) (Supplementary Material S11). Additionally, we controlled for potential effects driven by differences in sample size across countries by replicating the analysis using a bootstrap approach, where the sample of one country was removed at each step, completing a total of 20 cycles and 1200 combinations. Ridge regressions were conducted with 20 repetitions and 3-fold cross-validation, using the same controls and parameters as the previous analysis (Supplementary Material S12). Our main results were reproduced even in an age-matched subsample (Supplementary Material S13) or without adjustment for cognition (Supplementary Material S14).

Third, we tested for a potential effect of image quality using the SNR and tSNR values, where higher values indicate better quality. In the T1w sample, both EQ and YoE showed negative correlations with SNR (r_EQ_ = −0·15; r_YoE_ = −0·09), implying that higher EQ and YoE were associated with lower image quality. Nevertheless, we tested the effect of image quality on our results by removing SNR from the whole-brain volume measure using a general linear model. We then replicated the regression analyses, predicting this adjusted measure. After correction, our main results remained unaltered, with EQ being a better predictor than YoE (HCs: Δmean = 0·5 [0·4–0·6] × 10^−2^, *p* < 10^−5^; AD: Δmean = 7·4 [7·2–7·6] × 10^−2^, *p* < 10^−5^; FTLD: Δmean = 30·7 [30·1–31·4] × 10^−2^, *p* < 10^−5^, all with 95% confidence intervals). In the rs-fMRI sample, neither EQ nor YoE correlated with SNR (r_EQ_ = 0·07, r_YoE_ = 0·08) or tSNR (r_EQ_ = −0·02, r_YoE_ = −0·07). Additionally, after adjusting global efficiency using the same method to account for SNR and tSNR, EQ demonstrated an even higher prediction power that YoE (HCs: Δmean = 0·5 [0·3–0·7] × 10^−2^, *p* < 10^−5^; AD: Δmean = 3·4 [2·8–4·0] × 10^−2^, *p* < 10^−5^; FTLD: Δmean = 12·8 [11·8–13·7] × 10^−2^, *p* < 10^−5^).

## Discussion

This study compared the effect of EQ and YoE on brain structure and function across 20 countries. The findings showed that while both EQ and YoE are associated with reductions in gray matter volume and functional connectivity, EQ exerts a more substantial influence, even after accounting for age, sex, cognition and socioeconomic status. At the regional level, EQ affected frontal and parietal regions in HCs, had widespread effects in AD, and was mainly linked to frontotemporal areas in FTLD. EQ's influence was up to seven times greater than YoE at the whole-brain level. These results underline the more substantial role of education quality over quantity on brain health, offering a new perspective on country-level disparities. Our work highlights the need for future research on education disparities; and for public policies to improve access to education and the quality of education to promote brain health, particularly in underdeveloped countries.

Our results emphasize the value of aggregate measures over individual ones in brain associations. YoE reflects the duration of formal education but does not capture the cognitive skills embedded within an educational system. In contrast, EQ indicates the broader learning demands and skills required.^10^^,^^11^ For example, two individuals with similar educational attainment can develop different intellectual capacities depending on the quality of their education. Individuals with high cognitive potential may fail to reach their full intellectual capacity when living in a socially disadvantaged context.^22^ These capacities, developed during the educational process, enhance brain plasticity, potentially increasing resilience to brain damage.^1^ Moreover, measures focused on skills and the learning environment may offer complementary insights into brain health. Previous research has predominantly focused on individual-level factors, often overlooking the profound effect of environmental determinants.^6^^,^^8^^,^^9^^,^^23^ These environmental influences can be analyzed across multiple scales, from immediate surroundings to regional, national, and global levels. Country-level measures have been proven to influence brain volume and network organization beyond individual differences.^6^^,^^9^^,^^23^ Aggregate-level metrics tend to remain relatively stable over time,^24^^,^^25^ exerting a long-term influence on brain health. They contribute to cumulative effects across generations, shaping opportunities, access to resources, and social determinants of health. These factors may modulate brain health^2^^,^^3^^,^^6^^,^^9^ by exposing individuals to chronic stress,^26^ allostatic overload,^27^ and limited access to stimulating environments.^1^^,^^3^ These persistent influences create a macro-context that defines brain development and cognitive potential boundaries. By accumulating over time and across generations, aggregate factors can act as critical drivers of brain health disparities, surpassing individual factors.

EQ exerts differential influence across the studied groups, providing new insights into its role in brain health. Previous research has associated educational attainment and socioeconomic factors with temporal lobe and cerebellum damage.^3^^,^^23^ Our findings align with these observations but further reveal that EQ significantly influences specific vulnerable areas depending on the condition.^9^^,^^13^^,^^14^ The effect of EQ on brain health remains consistent across diverse environments, showing no dependence on country or individual socioeconomic differences. The gradient effect of EQ (AD > FTLD > HCs) suggests a hypothetical three-level model of genetics, pathology, and environmental factors. AD conveys genetic risk and neurodegeneration interacting with a cumulative education burden throughout life.^13^^,^^28^ These environmental factors are known to influence disease pathology,^28^ potentially accelerating neurodegeneration. This explains why individual and country-level effects can exhibit similar influences in this dementia phenotype. In contrast, FTLD has a more substantial genetic component less influenced by environmental factors than AD.^14^^,^^29^ Finally, HCs exhibit reduced genetic risk and no neurodegeneration, demonstrating a capacity to compensate for environmental stress.^30^ This compensatory ability may be reflected in fewer volume changes and a greater short-term network reorganization. More research is required to confirm these interpretations across groups.

Our study presents multiple limitations and calls for further research. First, PISA assesses mathematics, reading, and science, leaving out cognitive abilities such as creativity, problem-solving, and critical thinking. Also, PISA does not account for higher education or lifelong learning opportunities; however, it has been shown to influence access and success in both.^10^^,^^11^ Indeed, PISA provides a standardized measure of foundational skills strongly linked to later educational and cognitive outcomes, remaining the most widely accepted measure of EQ across countries.^10^^,^^11^ Further studies should assess broader aspects of education quality, including those outlined in the sustainable development goals and lifelong learning opportunities. Second, PISA measures the performance of 15-year-olds students, and its extrapolation to other age groups is supported by their stability over time. Also, it was compared to YoE, which is acquired early in life. Educational systems evolved gradually, with systemic educational disparities persisting for decades due to policy continuity, economic conditions, and structural inequalities. Studies have shown that countries with higher or lower educational performance today often had similar rankings historically, reinforcing the use of PISA as a valid approximation of past education quality.^10^^,^^11^ Our specific analysis (Supplementary Material S3) shows that PISA scores have remained stable from 2003 to 2022, indicating that national education systems maintain consistent relative performance over time. While no globally standardized metric exists to measure education quality in the 1960s–70s, this stability suggests that countries' relative educational rankings have remained unchanged for decades, making PISA the best proxy for cross-national comparisons. Moreover, before the introduction of the PISA, there were no standardized EQ scores across countries, making it impossible to obtain reliable EQ values for individuals over 25. Third, our cross-sectional study cannot establish causal relationships between EQ and brain alterations. Fourth, multicentric images can introduce variability, although these were controlled through harmonization pipelines and signal quality analyses (SNR, tSNR). Fifth, we lack data to isolate educational effect from other inequalities, such as cardiovascular health, nutrition, or socioeconomic disparities. Although our analysis included individual-level metrics (age, sex, cognition, and SES), aggregate-level metrics of EQ, by definition, do not capture intra-country variations.^5^ Future research should integrate longitudinal data, additional measures of disparities at the regional level, and advanced models to understand better the effect of educational disparities on brain health and dementia.

In conclusion, a greater effect of education quality over quantity on brain health was evidenced, highlighting a neglected research area that could inform policy recommendations to enhance brain health. Our results reveal distinct effects on healthy aging and dementia phenotypes, opening a new research agenda to assess the interaction of genetic, neurodegenerative, and environmental factors. By incorporating education quality into brain health research, this study offers a new framework to address global disparities in aging and dementia.

## Contributors

AI, RGG, and CCO led the study's conceptual design. RGG conducted the statistical analyses, and RGG, CCO, and AI collaboratively drafted the manuscript. All authors participated in interpreting the results, provided critical revisions, and approved the final version of the manuscript. RGG and CCO had complete access to the study data and verified its accuracy. All authors collectively decided to submit the manuscript for publication.

## Data sharing statement

Supplementary Table S1 provides the access details for each database used in this study. Pipelines will be made available upon reasonable request to the corresponding author.

## Declaration of interests

None to declare.
