# Supplementary material for: Qualitative and quantitative educational disparities and brain signatures in healthy aging and dementia across global settings
Source: eClinicalMedicine. 2025 Apr 10;82:103187. doi: 10.1016/j.eclinm.2025.103187 (PMC12018025; doi:10.1016/j.eclinm.2025.103187)
Supplement: Supplementary Figures and Tables [file mmc1.docx]

Table of contents

[S1: Data origin and sample size 2](#_Toc190289854)

[Supplementary Table S1. Data publication, origin, access, and sample size 2](#_Toc190289855)

[S2: Atrophy pattern in dementia phenotypes 3](#_Toc190289856)

[S3: Analysis of temporal changes in PISA scores within countries 3](#_Toc190289857)

[Supplementary Table S2. 3](#_Toc190289858)

[S4. Detailed statistics in atrophy analysis 4](#_Toc190289859)

[Supplementary Table S4. 4](#_Toc190289860)

[S5. Detailed statistics in network analysis 8](#_Toc190289861)

[Supplementary Table S6. 8](#_Toc190289862)

[S6. Size of the educational factors' effect on atrophy analyses 12](#_Toc190289863)

[Supplementary Table S3. 12](#_Toc190289864)

[S7. Size of the educational factors' effect on network analyses 12](#_Toc190289865)

[Supplementary Table S5. 12](#_Toc190289866)

[S8. Comparison of the effects of EQ and the Interaction (EQ × YoE) in regional atrophy analysis 13](#_Toc190289867)

[Supplementary Figure 2. 13](#_Toc190289868)

[S9. Comparison of the effects of EQ and the Interaction (EQ × YoE) in regional network analysis 14](#_Toc190289869)

[Supplementary Figure 3. 14](#_Toc190289870)

[S10. Comparison of the effects of EQ and the Interaction (EQ × YoE) in whole-brain analysis 14](#_Toc190289871)

[Supplementary Figure 4 15](#_Toc190289872)

[S11. Whole-brain analysis adjusted by income and socioeconomic values at country and individual levels 15](#_Toc190289873)

[Supplementary Figure 5 16](#_Toc190289874)

[S12. Whole-brain analysis adjusted by differences in sample size across countries 17](#_Toc190289875)

[Supplementary Figure 6. 17](#_Toc190289876)

[S13. Whole-brain analysis in groups matched by age 18](#_Toc190289877)

[Supplementary Figure 7. 18](#_Toc190289878)

[S14. Whole-brain analysis without adjustment by cognition 18](#_Toc190289879)

[Supplementary Figure 8 19](#_Toc190289880)

[References 20](#_Toc190289881)

# S1: Data origin and sample size

Supplementary Table S1 provides detailed information about the datasets employed, including their publication, origin, access, and sample size for each imaging modality (T1 and rs-fMRI) included in the study.

## Supplementary Table S1. Data publication, origin, access, and sample size

|  |  |  |  | sample size | | |  |
| --- | --- | --- | --- | --- | --- | --- | --- |
| Dataset | Country | Access | Modality | HCs | AD | FTLD |  |
| ADNI^1^ | USA | <https://ida.loni.usc.edu/> | T1w | 479 | 90 | - |  |
|  |  |  | fMRI | 455 | 75 |  |  |
| AIBL^2^ | Australia | <https://ida.loni.usc.edu/> | T1w | - | 655 | - |  |
| ARWIBO^3^ | Italy | <https://www.neugrid2.eu> | T1w | 852 | 186 | 33 |  |
| Cam-Can^4^ | United Kingdom | <https://camcan-archive.mrc-cbu.cam.ac.uk/> | T1w | 591 | - | - |  |
|  |  |  | fMRI | 533 |  |  |  |
| ds000148^5^ | Poland | [https://openneuro.org](https://openneuro.org/) | T1w | 49 | - | - |  |
| ds000201^6^ | Sweden | [https://openneuro.org](https://openneuro.org/) | T1w | 84 | - | - |  |
| ds002080^7^ | Belgium | [https://openneuro.org](https://openneuro.org/) | T1w | 10 | - | - |  |
| ds003592^8^ | Canada | [https://openneuro.org](https://openneuro.org/) | T1w | 63 | - | - |  |
| ds004557^9^ | Austria | [https://openneuro.org](https://openneuro.org/) | T1w | 35 | - | - |  |
| ds004711^10^ | Switzerland | [https://openneuro.org](https://openneuro.org/) | T1w | 182 | - | - |  |
| ds004894^11^ | Japon | [https://openneuro.org](https://openneuro.org/) | T1w | 49 | - | - |  |
| EDSD^3^ | Italy, Netherlands, Germany | <https://www.neugrid2.eu> | T1w | 161 | 170 | - |  |
|  |  |  |  |  |  |  |  |
| NIFD^12^ | USA | <https://ida.loni.usc.edu/> | T1w | 109 | - | 144 |  |
|  |  |  | fMRI | 102 | - | 221 |  |
| Gorsev^13^ | Turkey | Görsev Yener (gorsev.yener@deu.edu.tr) | T1w | 88 | 54 | - |  |
|  |  |  |  |  |  |  |  |
| HCP^14^ | USA | <https://www.humanconnectome.org/> | T1w | 808 | - | - |  |
|  |  |  | fMRI | 150 | - | - |  |
| I-ADNI^3^ | Italy | <https://www.neugrid2.eu> | T1w | 2 | 170 | 14 |  |
| Lemon^15^ | Germany | <https://fcon_1000.projects.nitrc.org/> | T1w | 226 | - | - |  |
|  |  |  | fMRI | 213 | - | - |  |
| ReDLat^16^ | Argentina, Brazil, Colombia, Chile, Mexico, Peru, USA | <https://red-lat.com/> | T1w | 932 | 793 | 467 |  |
|  |  |  | fMRI | 598 | 518 | 342 |  |
|  |  |  |  |  |  |  |  |
| VITA^3^ | Austria | <https://www.neugrid2.eu> | T1w | 37 | - | - |  |

Abbreviations: Alzheimer's disease, HCs: healthy controls, FTLD: frontotemporal lobar degeneration

# S2: Atrophy pattern in dementia phenotypes

The comparison of grey matter volume between HCs and dementia groups reveals well-established patterns of atrophy. In AD, the temporoposterior regions were primarily affected, with significant atrophy in the hippocampus (Supplementary Figure S1A).^17^ In contrast, FTLD predominantly impacts the anterior regions of the frontal and temporal lobes, with the most severe atrophy observed in the anterotemporal areas (Supplementary Figure S1B).^18^


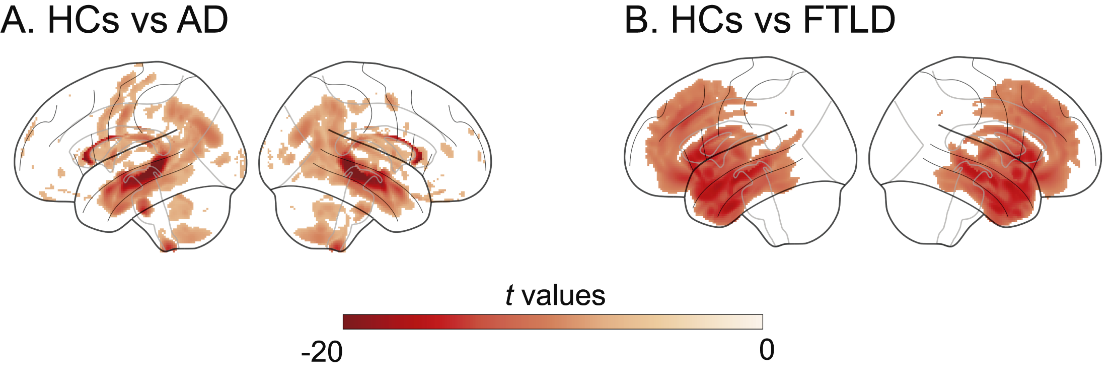


Supplementary Figure S1. Atrophy pattern in (A) Alzheimer's Disease (AD) and (B) Frontotemporal Lobar Degeneration (FTLD). Voxel-based morphometry was employed to compare healthy controls (HCs) with the respective dementia phenotypes. Results are presented as the brain maps of significative t-values corrected for multiple comparisons using a false discovery rate (FDR) at p < 0.001.

# S3: Analysis of temporal changes in PISA scores within countries

We used mixed linear model analysis to test changes over time in PISA scores within countries. The model predicted PISA scores using an interaction term (year × country) and a random effect for country (1|country). Results indicated no significant changes, with p-values exceeding 0·09. Detailed statistics and p-values for each country are provided in Supplementary Table S2.

Supplementary Table S2. Mixed linear model analysis of temporal changes in PISA scores within countries

| **Effect** | **estimate** | **std. error** | **statistic** | **p-value** |
| --- | --- | --- | --- | --- |
| year x Argentina | 0·010 | 0·026 | 0·388 | 0·699 |
| year x Australia | -0·009 | 0·024 | -0·370 | 0·712 |
| year x Austria | -0·006 | 0·024 | -0·232 | 0·817 |
| year x Belgium | -0·018 | 0·024 | -0·750 | 0·454 |
| year x Brazil | 0·007 | 0·024 | 0·293 | 0·770 |
| year x Canada | 0·013 | 0·024 | 0·556 | 0·579 |
| year x Chile | 0·005 | 0·026 | 0·174 | 0·862 |
| year x Colombia | 0·070 | 0·037 | 1·882 | 0·091 |
| year x Germany | 0·006 | 0·024 | 0·242 | 0·809 |
| year x Italy | 0·007 | 0·024 | 0·276 | 0·783 |
| year x Japan | 0·011 | 0·024 | 0·462 | 0·645 |
| year x Mexico | 0·015 | 0·024 | 0·633 | 0·527 |
| year x Netherlands | -0·003 | 0·024 | -0·119 | 0·906 |
| year x Peru | 0·052 | 0·029 | 1·783 | 0·096 |
| year x Poland | 0·003 | 0·024 | 0·132 | 0·895 |
| year x Sweden | -0·008 | 0·024 | -0·318 | 0·751 |
| year x Switzerland | 0·001 | 0·024 | 0·038 | 0·969 |
| year x Türkiye | 0·019 | 0·024 | 0·779 | 0·437 |
| year x United Kingdom | -0·021 | 0·024 | -0·864 | 0·388 |
| year x United States | 0·007 | 0·024 | 0·296 | 0·768 |

Abbreviations: std: standard.

# S4. Detailed statistics in atrophy analysis

Supplementary Table 4 presents the significant clusters and their peak values for each effect across groups, offering a detailed spatial characterization of the atrophy patterns linked to EQ and YoE.

Supplementary Table S4. Detailed statistics in atrophy analysis

| **Cluster** | **No. voxels** | **Region** | **Peak R^2^ Value** | **Peak p-value** |
| --- | --- | --- | --- | --- |
| **HCs_PISA** | | | | |
| 1 | 10182 | Left Parietal Lobe | 0·24 | 1E-247 |
| 2 | 33281 | Right Frontal Lobe | 0·27 | 1E-276 |
| 3 | 2134 | Right Parietal Lobe | 0·17 | 1E-169 |
| 4 | 211 | Right Temporal/Occipital Lobe | 0·10 | 3E-92 |
| 5 | 1841 | Cerebellum | 0·18 | 8E-199 |
| 6 | 458 | Right Temporal/Occipital Lobe | 0·10 | 4E-74 |
| 7 | 3010 | Right Frontal Lobe | 0·24 | 1E-186 |
| 8 | 419 | Right Frontal Lobe | 0·15 | 4E-150 |
| 9 | 693 | Right Parietal Lobe | 0·12 | 3E-110 |
| 10 | 776 | Right Parietal Lobe | 0·12 | 1E-94 |
| 11 | 303 | Right Frontal Lobe | 0·14 | 1E-144 |
| 12 | 753 | Right Parietal Lobe | 0·13 | 1E-135 |
| 13 | 194 | Left Parietal Lobe | 0·15 | 4E-92 |
| 14 | 3383 | Right Temporal/Occipital Lobe | 0·15 | 1E-134 |
| 15 | 129 | Right Frontal Lobe | 0·10 | 8E-82 |
| 16 | 516 | Left Temporal/Occipital Lobe | 0·23 | 6E-222 |
| 17 | 564 | Right Frontal Lobe | 0·11 | 3E-58 |
| 18 | 62 | Left Parietal Lobe | 0·10 | 1E-106 |
| 19 | 6313 | Left Temporal/Occipital Lobe | 0·23 | 1E-234 |
| 20 | 2257 | Left Temporal/Occipital Lobe | 0·18 | 1E-200 |
| 21 | 615 | Left Frontal Lobe | 0·10 | 1E-82 |
| 22 | 4518 | Left Parietal Lobe | 0·19 | 2E-198 |
| 23 | 1141 | Left Parietal Lobe | 0·12 | 4E-85 |
| 24 | 69 | Left Parietal Lobe | 0·09 | 3E-60 |
| 25 | 434 | Left Frontal Lobe | 0·14 | 3E-143 |
| 26 | 475 | Left Parietal Lobe | 0·13 | 4E-121 |
| 27 | 3574 | Left Parietal Lobe | 0·14 | 3E-130 |
| 28 | 84 | Left Temporal/Occipital Lobe | 0·10 | 1E-80 |
| 29 | 90 | Right Temporal/Occipital Lobe | 0·09 | 1E-84 |
| HCs_YoE | | | | |
| 1 | 2321 | Left Parietal Lobe | 0·14 | 3E-145 |
| 2 | 26548 | Right Parietal Lobe | 0·26 | 3E-246 |
| 3 | 4502 | Right Parietal Lobe | 0·21 | 5E-224 |
| 4 | 248 | Right Frontal Lobe | 0·12 | 6E-118 |
| 5 | 1595 | Right Temporal/Occipital Lobe | 0·16 | 1E-160 |
| 6 | 1113 | Cerebellum | 0·18 | 8E-196 |
| 7 | 67 | Right Temporal/Occipital Lobe | 0·09 | 7E-72 |
| 8 | 149 | Right Frontal Lobe | 0·09 | 2E-70 |
| 9 | 277 | Right Frontal Lobe | 0·14 | 6E-135 |
| 10 | 148 | Right Parietal Lobe | 0·10 | 5E-100 |
| 11 | 77 | Right Parietal Lobe | 0·10 | 9E-81 |
| 12 | 212 | Right Frontal Lobe | 0·12 | 2E-132 |
| 13 | 487 | Left Parietal Lobe | 0·13 | 7E-131 |
| 14 | 784 | Left Parietal Lobe | 0·12 | 7E-117 |
| 15 | 739 | Left Frontal Lobe | 0·11 | 5E-106 |
| 16 | 9023 | Right Frontal Lobe | 0·26 | 1E-274 |
| 17 | 455 | Right Frontal Lobe | 0·21 | 1E-207 |
| 18 | 50 | Left Parietal Lobe | 0·10 | 2E-100 |
| 19 | 4608 | Left Temporal/Occipital Lobe | 0·21 | 9E-221 |
| 20 | 1179 | Left Temporal/Occipital Lobe | 0·18 | 1E-197 |
| 21 | 519 | Left Frontal Lobe | 0·10 | 2E-85 |
| 22 | 852 | Left Frontal Lobe | 0·15 | 3E-160 |
| 23 | 631 | Left Parietal Lobe | 0·11 | 3E-110 |
| 24 | 278 | Left Frontal Lobe | 0·13 | 7E-133 |
| 25 | 269 | Left Frontal Lobe | 0·11 | 2E-107 |
| 26 | 2177 | Left Temporal/Occipital Lobe | 0·19 | 9E-196 |
| 27 | 1977 | Left Parietal Lobe | 0·12 | 1E-120 |
| 28 | 87 | Left Temporal/Occipital Lobe | 0·10 | 1E-88 |
| AD_PISA | | | |  |
| 1 | 295684 | Right Parietal Lobe | 0·33 | 1E-78 |
|  |  | Frontal lobe | 0·17 | 4E-16 |
|  |  | Temporal lobe | 0·28 | 3E-46 |
|  |  | Parietal lobe | 0·13 | 4E-13 |
|  |  | Occipital | 0·17 | 4E-16 |
|  |  | Cerebellum | 0·19 | 5E-23 |
| 2 | 118 | Left Frontal Lobe | 0·13 | 9E-19 |
| 3 | 56 | Right Temporal/Occipital Lobe | 0·13 | 3E-44 |
| AD_YoE | | | | |
| 1 | 5565 | Right Parietal Lobe | 0·28 | 6E-07 |
| 2 | 520 | Right Frontal Lobe | 0·16 | 1E-05 |
| 3 | 56 | Right Parietal Lobe | 0·12 | 1E-03 |
| 4 | 114 | Cerebellum | 0·14 | 8E-03 |
| 5 | 119 | Right Frontal Lobe | 0·14 | 9E-03 |
| 6 | 128 | Right Parietal Lobe | 0·10 | 2E-03 |
| 7 | 415 | Right Frontal Lobe | 0·15 | 4E-04 |
| 8 | 162 | Cerebellum | 0·16 | 2E-03 |
| 9 | 2484 | Right Frontal Lobe | 0·19 | 8E-04 |
| 10 | 110 | Right Temporal/Occipital Lobe | 0·14 | 3E-03 |
| 11 | 51 | Right Parietal Lobe | 0·11 | 5E-03 |
| 12 | 330 | Right Frontal Lobe | 0·13 | 4E-05 |
| 13 | 129 | Right Parietal Lobe | 0·14 | 1E-03 |
| 14 | 63 | Right Frontal Lobe | 0·13 | 1E-02 |
| 15 | 81 | Right Frontal Lobe | 0·23 | 5E-03 |
| 16 | 455 | Right Parietal Lobe | 0·12 | 1E-04 |
| 17 | 181 | Left Temporal/Occipital Lobe | 0·13 | 6E-07 |
| 18 | 14672 | Right Parietal Lobe | 0·29 | 3E-21 |
| 19 | 1403 | Right Parietal Lobe | 0·18 | 5E-04 |
| 20 | 696 | Right Temporal/Occipital Lobe | 0·19 | 1E-03 |
| 21 | 56 | Right Parietal Lobe | 0·12 | 8E-11 |
| 22 | 67 | Right Parietal Lobe | 0·13 | 8E-05 |
| 23 | 127 | Right Frontal Lobe | 0·12 | 3E-04 |
| 24 | 411 | Left Temporal/Occipital Lobe | 0·15 | 1E-03 |
| 25 | 339 | Right Temporal/Occipital Lobe | 0·14 | 1E-09 |
| 26 | 136 | Right Frontal Lobe | 0·10 | 3E-27 |
| 27 | 275 | Left Temporal/Occipital Lobe | 0·12 | 3E-04 |
| 28 | 440 | Left Frontal Lobe | 0·10 | 9E-27 |
| 29 | 179 | Left Parietal Lobe | 0·23 | 4E-03 |
| 30 | 588 | Left Frontal Lobe | 0·21 | 4E-05 |
| 31 | 300 | Cerebellum | 0·17 | 6E-04 |
| 32 | 241 | Left Parietal Lobe | 0·10 | 4E-27 |
| 33 | 194 | Left Temporal/Occipital Lobe | 0·14 | 2E-06 |
| 34 | 211 | Left Frontal Lobe | 0·15 | 9E-03 |
| 35 | 418 | Left Parietal Lobe | 0·19 | 6E-04 |
| 36 | 281 | Left Temporal/Occipital Lobe | 0·17 | 1E-03 |
| 37 | 4278 | Left Frontal Lobe | 0·20 | 1E-04 |
| 38 | 726 | Left Frontal Lobe | 0·17 | 2E-04 |
| 39 | 252 | Left Parietal Lobe | 0·16 | 1E-04 |
| 40 | 50 | Left Parietal Lobe | 0·09 | 5E-05 |
| 41 | 92 | Cerebellum | 0·15 | 1E-03 |
| 42 | 58 | Left Temporal/Occipital Lobe | 0·15 | 8E-03 |
| 43 | 83 | Left Parietal Lobe | 0·14 | 8E-03 |
| 44 | 91 | Left Parietal Lobe | 0·21 | 1E-02 |
| 45 | 214 | Left Frontal Lobe | 0·15 | 8E-04 |
| 46 | 109 | Left Frontal Lobe | 0·14 | 2E-03 |
| 47 | 53 | Left Parietal Lobe | 0·11 | 2E-03 |
| 48 | 52 | Right Parietal Lobe | 0·11 | 7E-03 |
| FTLD_PISA | | | | |
| 1 | 4998 | Right Parietal Lobe | 0·13 | 3E-11 |
| 2 | 370 | Right Parietal Lobe | 0·10 | 4E-05 |
| 3 | 220 | Right Frontal Lobe | 0·10 | 3E-08 |
| 9 | 212 | Right Parietal Lobe | 0·10 | 3E-03 |
| 16 | 11318 | Right Temporal Lobe | 0·25 | 6E-12 |
| 26 | 107 | Right Parietal Lobe | 0·09 | 3E-09 |
| 29 | 115 | Cerebellum | 0·10 | 1E-05 |
| 41 | 30994 | Right Parietal Lobe | 0·21 | 2E-12 |
| 47 | 275 | Right Frontal Lobe | 0·20 | 2E-07 |
| 48 | 620 | Right Parietal Lobe | 0·15 | 4E-07 |
| 67 | 142 | Cerebellum | 0·09 | 2E-11 |
| 73 | 539 | Right Parietal Lobe | 0·11 | 7E-11 |
| 75 | 75 | Right Temporal/Occipital Lobe | 0·11 | 5E-06 |
| 78 | 90 | Left Parietal Lobe | 0·13 | 5E-07 |
| 86 | 354 | Left Frontal Lobe | 0·20 | 3E-05 |
| 88 | 494 | Left Temporal/Occipital Lobe | 0·12 | 2E-03 |
| 98 | 1093 | Left Frontal Lobe | 0·10 | 2E-10 |
| 103 | 1008 | Left Frontal Lobe | 0·20 | 5E-07 |
| 105 | 236 | Cerebellum | 0·12 | 2E-04 |
| 112 | 298 | Left Parietal Lobe | 0·24 | 8E-08 |
| 118 | 1489 | Left Parietal Lobe | 0·14 | 1E-06 |
| 133 | 650 | Left Parietal Lobe | 0·12 | 2E-04 |
| 142 | 2277 | Left Frontal Lobe | 0·12 | 4E-09 |
| 145 | 129 | Left Frontal Lobe | 0·14 | 7E-03 |
| 153 | 125 | Left Frontal Lobe | 0·13 | 2E-03 |
| 155 | 93 | Left Frontal Lobe | 0·11 | 3E-03 |
| 159 | 207 | Right Temporal/Occipital Lobe | 0·12 | 2E-03 |
| FTLD_YoE | | | | |
| 1 | 773 | Right Temporal/Occipital Lobe | 0·12 | 7E-09 |
| 2 | 661 | Left Temporal/Occipital Lobe | 0·13 | 2E-03 |
| 3 | 10337 | Right Temporal/Occipital Lobe | 0·15 | 5E-14 |
| 4 | 101 | Left Frontal Lobe | 0·10 | 3E-12 |
| 5 | 54 | Cerebellum | 0·08 | 1E-05 |
| 6 | 104 | Left Temporal/Occipital Lobe | 0·10 | 6E-03 |
| 7 | 53 | Left Frontal Lobe | 0·11 | 1E-13 |
| 8 | 271 | Left Parietal Lobe | 0·09 | 2E-05 |

# S5. Detailed statistics in network analysis

Supplementary Table 6 presents the significant connections exceeding 70% and their R^2^ values for each effect across groups, offering a detailed spatial characterization of the functional changes linked to EQ and YoE.

Supplementary Table S6. Detailed statistics in network analysis

| **Connection** | **R^2^** | **p-value** |
| --- | --- | --- |
| **HCs_PISA** | | |
| Frontal Sup L - Rectus L | 0·23 | 4E-16 |
| Frontal Mid L - Frontal Mid Orb L | 0·20 | 6E-03 |
| Rolandic Oper L - Heschl R | 0·24 | 4E-16 |
| Rolandic Oper L - Temporal Sup L | 0·25 | 3E-09 |
| Rolandic Oper R - Heschl L | 0·20 | 2E-24 |
| Rolandic Oper R - Heschl R | 0·22 | 3E-23 |
| Rolandic Oper R - Temporal Sup L | 0·20 | 3E-19 |
| Frontal Sup Med L - Rectus L | 0·27 | 9E-16 |
| Frontal Sup Med L - Rectus R | 0·23 | 2E-13 |
| Frontal Sup Med R - Rectus L | 0·22 | 3E-11 |
| Frontal Sup Med R - Rectus R | 0·23 | 4E-13 |
| Frontal Med Orb L - Rectus L | 0·24 | 2E-21 |
| Frontal Med Orb L - Cingulum Post L | 0·19 | 4E-18 |
| Frontal Med Orb R - Cingulum Post L | 0·21 | 3E-15 |
| Frontal Med Orb R - Cingulum Post R | 0·20 | 1E-11 |
| Rectus L - Cingulum Post L | 0·26 | 6E-21 |
| Rectus L - Angular L | 0·26 | 3E-25 |
| Rectus R - Cingulum Post L | 0·26 | 3E-22 |
| Rectus R - Cingulum Post R | 0·21 | 4E-11 |
| Rectus R - Angular L | 0·25 | 3E-21 |
| Rectus R - Angular R | 0·22 | 2E-16 |
| Insula L - Thalamus L | 0·20 | 3E-02 |
| Insula L - Thalamus R | 0·19 | 9E-03 |
| Insula L - Cerebelum 8 L | 0·21 | 1E-16 |
| Insula R - Thalamus L | 0·27 | 1E-04 |
| Insula R - Thalamus R | 0·27 | 7E-08 |
| Cingulum Mid L - Cingulum Post R | 0·21 | 7E-03 |
| Cingulum Mid L - Pallidum L | 0·23 | 1E-03 |
| Cingulum Mid L - Cerebelum 9 L | 0·20 | 3E-09 |
| Cingulum Mid L - Cerebelum 9 R | 0·22 | 3E-15 |
| Cingulum Mid R - Pallidum L | 0·21 | 5E-04 |
| Cingulum Mid R - Pallidum R | 0·20 | 6E-11 |
| Cingulum Mid R - Cerebelum 9 L | 0·22 | 2E-10 |
| Hippocampus L - Hippocampus R | 0·21 | 7E-18 |
| SupraMarginal R - Cerebelum 8 L | 0·20 | 4E-10 |
| Angular R - Cerebelum 9 L | 0·21 | 5E-09 |
| Precuneus L - Cerebelum 9 L | 0·21 | 2E-04 |
| Precuneus L - Cerebelum 9 R | 0·22 | 8E-07 |
| Precuneus L - Cerebelum 10 R | 0·21 | 2E-11 |
| Precuneus R - Cerebelum 9 L | 0·22 | 5E-05 |
| Putamen L - Putamen R | 0·24 | 7E-14 |
| Putamen L - Pallidum R | 0·25 | 2E-18 |
| Putamen L - Cerebelum 6 L | 0·19 | 1E-07 |
| Putamen R - Pallidum R | 0·21 | 5E-26 |
| Putamen R - Cerebelum 6 L | 0·21 | 4E-11 |
| Pallidum L - Pallidum R | 0·22 | 9E-10 |
| Pallidum L - Cerebelum 6 L | 0·22 | 1E-09 |
| Pallidum L - Cerebelum 6 R | 0·22 | 1E-10 |
| Pallidum L - Cerebelum 8 L | 0·23 | 4E-06 |
| Pallidum L - Cerebelum 8 R | 0·22 | 1E-06 |
| Pallidum L - Cerebelum 9 L | 0·23 | 3E-11 |
| Pallidum L - Cerebelum 9 R | 0·25 | 2E-17 |
| Pallidum L - Vermis 8 | 0·19 | 2E-02 |
| Pallidum R - Cerebelum 4 5 L | 0·23 | 5E-06 |
| Pallidum R - Cerebelum 6 L | 0·26 | 2E-14 |
| Pallidum R - Cerebelum 6 R | 0·21 | 2E-09 |
| Pallidum R - Cerebelum 8 L | 0·22 | 1E-15 |
| Pallidum R - Cerebelum 9 L | 0·21 | 1E-15 |
| Thalamus R - Heschl L | 0·20 | 3E-18 |
| **HCs_YoE** | | |
| Precentral L - Heschl R | 0·15 | 2E-37 |
| Precentral R - Heschl R | 0·14 | 1E-36 |
| Frontal Inf Oper R - Heschl R | 0·14 | 5E-09 |
| Rolandic Oper L - Heschl L | 0·14 | 3E-16 |
| Rolandic Oper L - Heschl R | 0·14 | 7E-21 |
| Rolandic Oper R - Heschl L | 0·15 | 5E-29 |
| Rolandic Oper R - Heschl R | 0·15 | 4E-28 |
| Frontal Sup Med L - Rectus L | 0·13 | 1E-20 |
| Frontal Sup Med L - Temporal Pol Mid R | 0·14 | 2E-37 |
| Frontal Sup Med R - Temporal Pol Mid R | 0·13 | 2E-34 |
| Frontal Med Orb L - Rectus L | 0·14 | 2E-26 |
| Rectus L - Angular L | 0·13 | 2E-30 |
| Rectus L - Temporal Pol Mid L | 0·15 | 1E-24 |
| Rectus L - Temporal Pol Mid R | 0·14 | 3E-23 |
| Rectus R - Temporal Pol Mid R | 0·13 | 5E-17 |
| Hippocampus L - Hippocampus R | 0·18 | 1E-21 |
| Amygdala L - Amygdala R | 0·17 | 3E-21 |
| Occipital Mid L - Postcentral R | 0·14 | 2E-02 |
| Occipital Inf L - Heschl R | 0·14 | 7E-13 |
| Fusiform L - Heschl R | 0·16 | 1E-21 |
| Fusiform R - Heschl R | 0·14 | 2E-19 |
| Postcentral L - Heschl R | 0·13 | 1E-36 |
| Precuneus L - Cerebelum 10 R | 0·13 | 3E-15 |
| Putamen L - Pallidum R | 0·15 | 3E-23 |
| Putamen R - Pallidum R | 0·14 | 8E-31 |
| Pallidum L - Cerebelum 9 R | 0·13 | 3E-22 |
| Thalamus L - Heschl R | 0·12 | 1E-17 |
| Thalamus R - Heschl R | 0·14 | 3E-19 |
| Heschl R - Temporal Sup L | 0·14 | 9E-22 |
| Heschl R - Temporal Sup R | 0·13 | 1E-11 |
| Heschl R - Temporal Mid R | 0·13 | 1E-05 |
| **AD_PISA** | | |
| Precentral L - Insula L | 0·10 | 2E-02 |
| Precentral L - Insula R | 0·09 | 5E-02 |
| Precentral L - Precuneus L | 0·07 | 6E-03 |
| Frontal Inf Oper L - Insula R | 0·10 | 8E-03 |
| Rolandic Oper L - Supp Motor Area L | 0·08 | 4E-02 |
| Rolandic Oper R - Supp Motor Area L | 0·07 | 4E-02 |
| Supp Motor Area L - Insula L | 0·15 | 6E-03 |
| Supp Motor Area L - Insula R | 0·16 | 6E-03 |
| Supp Motor Area L - Temporal Pole Sup R | 0·11 | 3E-02 |
| Supp Motor Area R - Insula L | 0·13 | 6E-03 |
| Supp Motor Area R - Insula R | 0·14 | 6E-03 |
| Supp Motor Area R - Temporal Sup L | 0·08 | 5E-02 |
| Supp Motor Area R - Temporal Pole Sup L | 0·11 | 6E-03 |
| Supp Motor Area R - Temporal Pole Sup R | 0·14 | 1E-02 |
| Insula L - Cingulum Mid L | 0·11 | 4E-02 |
| Insula R - Cingulum Mid L | 0·13 | 2E-02 |
| Insula R - Cingulum Mid R | 0·13 | 8E-03 |
| Insula R - Heschl R | 0·07 | 5E-02 |
| Cingulum Mid L - Heschl L | 0·08 | 3E-02 |
| Cingulum Mid L - Heschl R | 0·08 | 4E-02 |
| Cingulum Mid R - Temporal Pole Sup L | 0·12 | 4E-02 |
| Parietal Inf L - Precuneus L | 0·06 | 4E-02 |
| Temporal Pole Sup R - Vermis 10 | 0·05 | 4E-02 |
| Cerebelum Crus2 R - Cerebelum 8 R | 0·05 | 4E-02 |
| Cerebelum 6 L - Vermis 9 | 0·05 | 4E-02 |
| Cerebelum 9 L - Cerebelum 9 R | 0·05 | 1E-02 |
| **AD_YoE** | | |
| Rolandic Oper L - Supp Motor Area L | 0·05 | 3E-02 |
| Rolandic Oper R - Supp Motor Area L | 0·06 | 3E-02 |
| Rolandic Oper R - Supp Motor Area R | 0·06 | 5E-02 |
| Supp Motor Area L - Insula L | 0·10 | 3E-03 |
| Supp Motor Area L - Insula R | 0·10 | 3E-03 |
| Supp Motor Area L - Temporal Pole Sup L | 0·06 | 3E-02 |
| Supp Motor Area L - Temporal Pole Sup R | 0·09 | 2E-02 |
| Supp Motor Area R - Insula L | 0·09 | 3E-03 |
| Supp Motor Area R - Insula R | 0·09 | 3E-03 |
| Rectus L - Temporal Pol Mid L | 0·06 | 4E-02 |
| Insula L - Cingulum Mid L | 0·06 | 2E-02 |
| Insula R - Cingulum Mid L | 0·08 | 2E-02 |
| Insula R - Cingulum Mid R | 0·08 | 3E-03 |
| Cingulum Ant R - Heschl R | 0·06 | 3E-02 |
| Cingulum Mid L - Heschl L | 0·05 | 1E-02 |
| Cingulum Mid L - Temporal Pole Sup L | 0·06 | 3E-02 |
| Cingulum Mid R - Temporal Pole Sup L | 0·06 | 2E-02 |
| Cerebelum Crus2 R - Cerebelum 8 R | 0·05 | 2E-02 |
| Cerebelum 6 L - Vermis 9 | 0·06 | 2E-02 |
| **FTLD_PISA** | | |
| Frontal Inf Tri L - Cingulum Mid R | 0·05 | 4E-02 |
| Frontal Inf Tri L - Postcentral L | 0·05 | 4E-02 |
| Frontal Sup Med R - Cingulum Ant L | 0·14 | 4E-02 |
| Frontal Sup Med R - Vermis 6 | 0·05 | 4E-02 |
| Frontal Med Orb L - Vermis 6 | 0·05 | 4E-02 |
| Cingulum Post L - Occipital Mid L | 0·05 | 5E-02 |
| Cingulum Post R - Putamen L | 0·05 | 4E-02 |
| Cingulum Post R - Temporal Mid L | 0·07 | 5E-02 |
| Cingulum Post R - Temporal Inf L | 0·08 | 5E-02 |
| Amygdala L - Heschl R | 0·09 | 1E-03 |
| Calcarine L - Putamen L | 0·05 | 5E-02 |
| Calcarine L - Putamen R | 0·06 | 5E-02 |
| Cuneus L - Putamen L | 0·06 | 5E-02 |
| Fusiform R - Cerebelum 4 5 L | 0·07 | 5E-02 |
| Postcentral R - Putamen R | 0·06 | 5E-02 |
| Paracentral Lob L - Putamen L | 0·06 | 5E-02 |
| Paracentral Lob R - Putamen L | 0·06 | 5E-02 |
| Thalamus R - Cerebelum Crus1 R | 0·07 | 5E-02 |
| **FTLD_YoE** | | |
| Frontal Mid Orb L - Cerebelum 4 5 L | 0·05 | 4E-02 |
| Rolandic Oper R - Heschl R | 0·06 | 4E-02 |
| Rolandic Oper R - Temporal Sup L | 0·06 | 4E-02 |
| Frontal Sup Med R - Cingulum Ant L | 0·08 | 4E-02 |
| Frontal Sup Med R - Cerebelum 8 L | 0·05 | 4E-02 |

# S6. Size of the educational factors' effect on atrophy analyses

To summarize the size of each effect, we calculated the total number of significant voxels across groups, along with the mean and range of R² values. These results are detailed in Supplementary Table 3.

Supplementary Table S3. Size of the educational factors' effect on atrophy analyses

| Gpo | Factor | Number of Significant Voxels | Mean R^2^ | Min R^2^ | Max R^2^ |  |
| --- | --- | --- | --- | --- | --- | --- |
|  |  |  |  |  |  |  |
| HCs | YoE | 39893 | 0·11 | 0·05 | 0·24 |  |
| HCs | EQ | 78976 | 0·12 | 0·05 | 0·27 |  |
| HCs | YoE x EQ | 72523 | 0·11 | 0·05 | 0·27 |  |
| AD | YoE | 39407 | 0·14 | 0·05 | 0·29 |  |
| AD | EQ | 295931 | 0·13 | 0·05 | 0·33 |  |
| AD | YoE x EQ | 293118 | 0·13 | 0·05 | 0·32 |  |
| FTLD | YoE | 12780 | 0·10 | 0·05 | 0·15 |  |
| FTLD | EQ | 59419 | 0·10 | 0·05 | 0·26 |  |
| FTLD | YoE x EQ | 48830 | 0·10 | 0·05 | 0·21 |  |

# S7. Size of the educational factors' effect on network analyses

To summarize the size of each effect, we calculated the total number of significant connections across groups, along with the mean and range of R² values. These results are detailed in Supplementary Table 5.

Supplementary Table S5. Size of the education effect on network analyses

| Gpo | Factor | Number of Significant connections | Mean R^2^ | Min R^2^ | Max R^2^ |  |
| --- | --- | --- | --- | --- | --- | --- |
|  |  |  |  |  |  |  |
| HCs | EQ | 2384 | 0·10 | 0·05 | 0·27 |  |
| HCs | YoE | 1136 | 0·07 | 0·05 | 0·18 |  |
| HCs | EQ x YoE | 2077 | 0·08 | 0·05 | 0·21 |  |
| AD | EQ | 30 | 0·09 | 0·05 | 0·16 |  |
| AD | YoE | 19 | 0·07 | 0·05 | 0·10 |  |
| AD | EQ x YoE | 20 | 0·10 | 0·05 | 0·14 |  |
| FTLD | EQ | 26 | 0·07 | 0·05 | 0·14 |  |
| FTLD | YoE | 5 | 0·07 | 0·05 | 0·08 |  |
| FTLD | EQ x YoE | 16 | 0·06 | 0·05 | 0·06 |  |

# S8. Comparison of the effects of EQ and the Interaction (EQ × YoE) in regional atrophy analysis

Supplementary Figure 2 displays the effect maps for each predictor and their differences across groups.


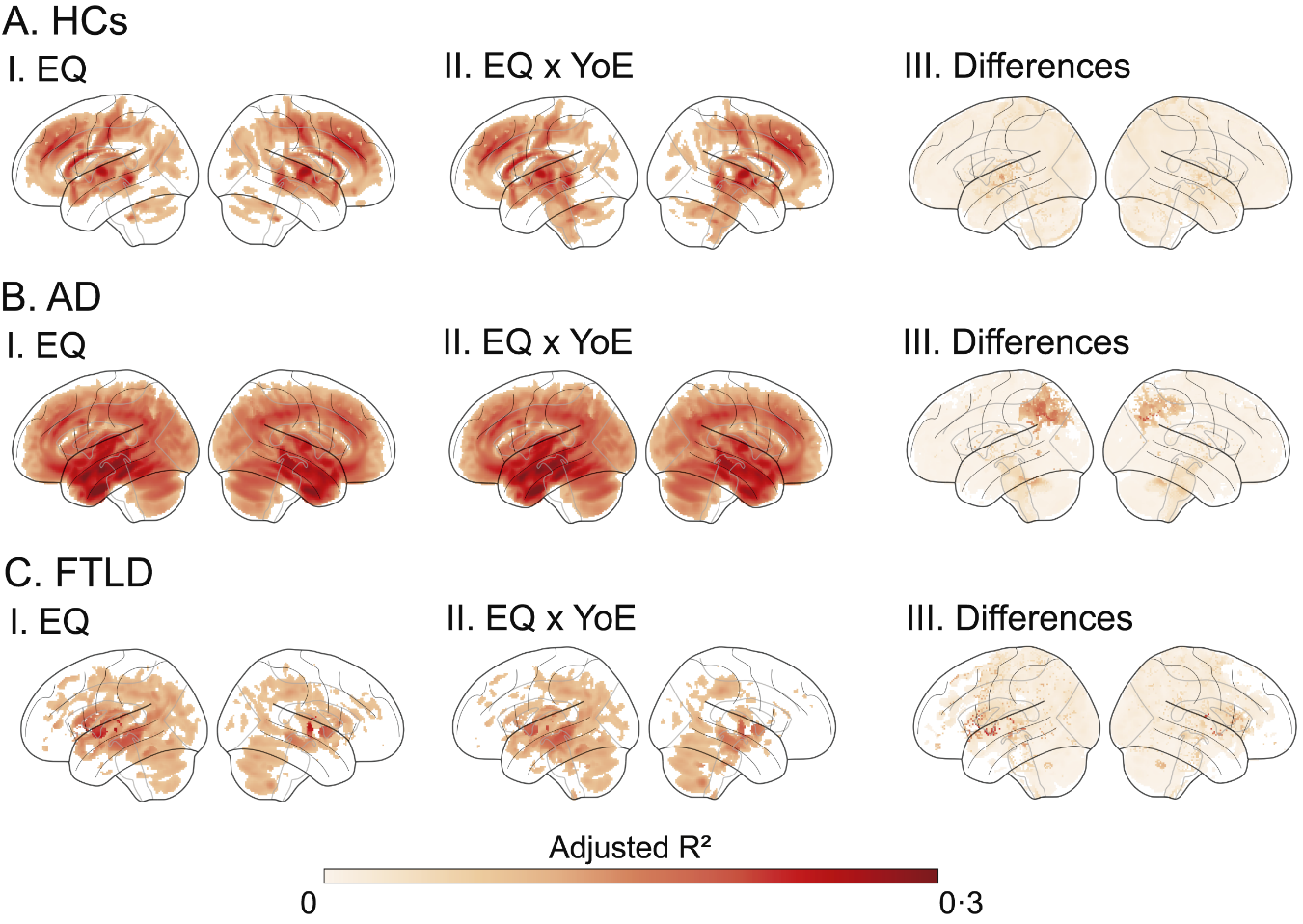


Supplementary Figure 2. Impact of education on voxel-wise gray matter volume. Analyses were conducted separately for **(A)** healthy controls, **(B)** Alzheimer's disease, and **(C)** frontotemporal lobar degeneration groups. Voxel-wise gray matter volume was predicted using ordinary least squares regression based on (I) Education Quality (EQ) or (II) the interaction of EQ and years of education (EQ x YoE), with age, sex, and cognition as covariates. Multiple corrections were applied using the False Discovery Rate (p_FDR_ < 0·05). Additionally, panel III presents the adjusted R^2^ difference between EQ and EQ x YoE predictions (I - II). *Abbreviations*: AD: Alzheimer's disease, EQ: education quality, FTLD: frontotemporal lobar degeneration, HCs: healthy controls, YoE: years of education.

# S9. Comparison of the effects of EQ and the Interaction (EQ × YoE) in regional network analysis

Supplementary Figure 3 displays the effect maps for each predictor and their differences across groups.


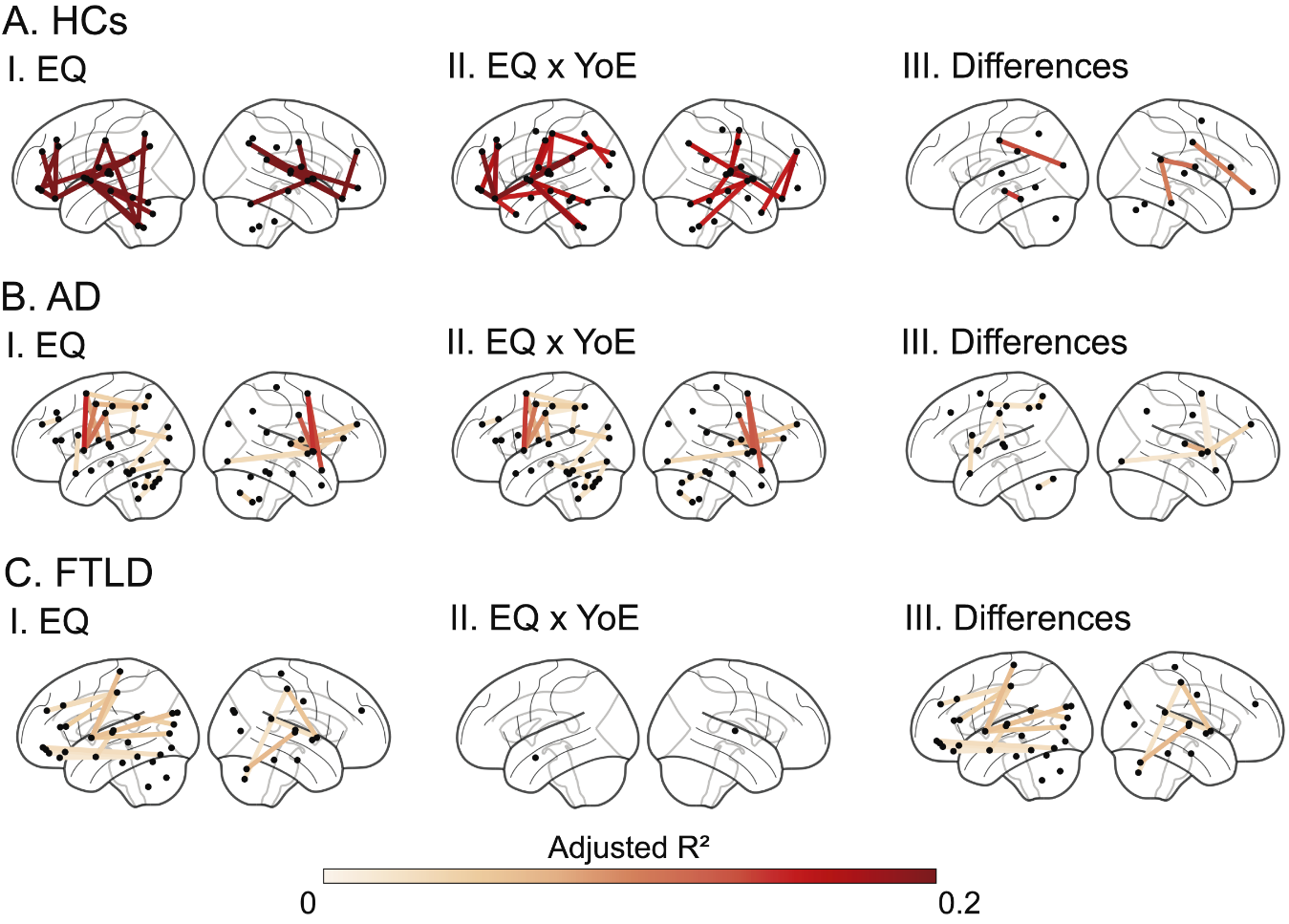


Supplementary Figure 3. Impact of education on pairwise functional connectivity. Analyses were conducted separately for **(A)** healthy controls, **(B)** Alzheimer's disease, and **(C)** frontotemporal lobar degeneration groups. Voxel-wise gray matter volume was predicted using ordinary least squares regression based on (I) Education Quality (EQ) or (II) the interaction of EQ and years of education (EQ x YoE), with age, sex, and cognition as covariates. Multiple corrections were applied using the False Discovery Rate (p_FDR_ < 0·05). Additionally, panel III presents the adjusted R^2^ difference between EQ and EQ x YoE predictions (I - II). *Abbreviations*: AD: Alzheimer's disease, EQ: education quality, FTLD: frontotemporal lobar degeneration, HCs: healthy controls, YoE: years of education.

# S10. Comparison of the effects of EQ and the Interaction (EQ × YoE) in whole-brain analysis

Supplementary Figure 4 displays the ridge regression analysis for each modality across predictors and groups.


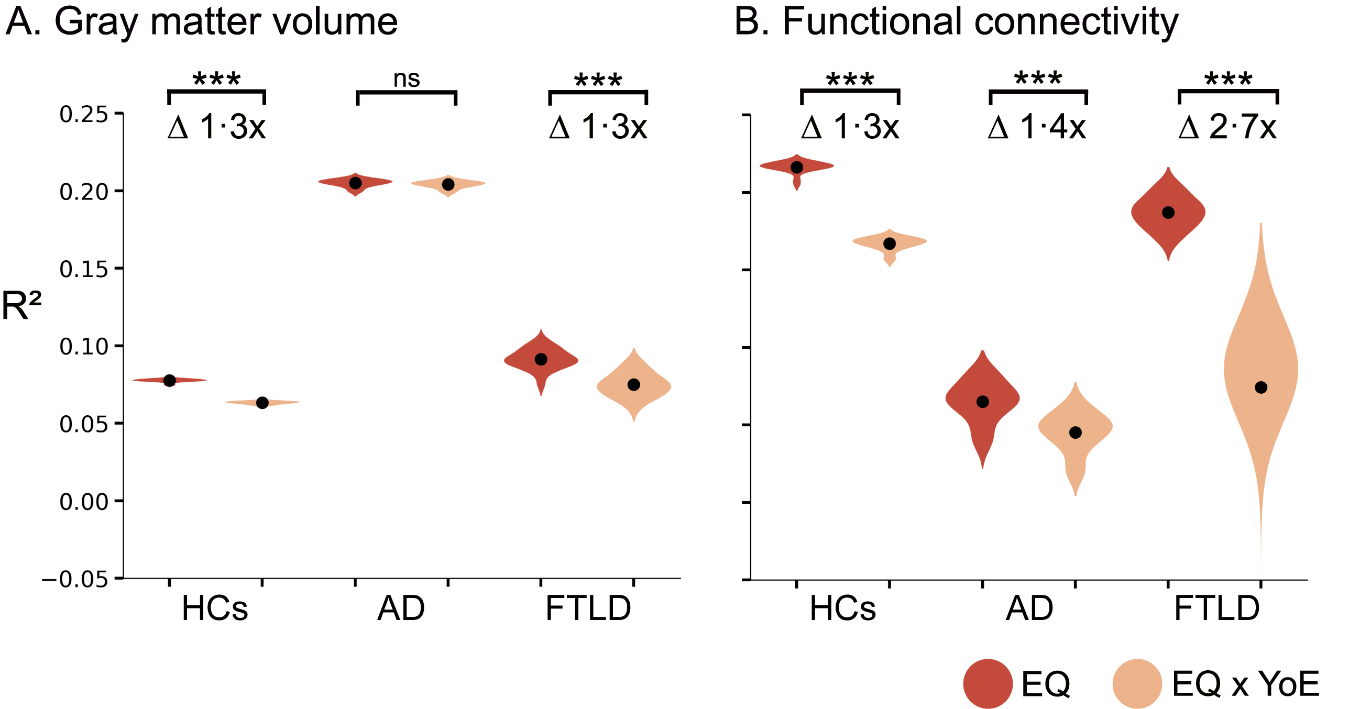


Supplementary Figure 4**.** Impact of education on whole-brain measures of (**A**) grey matter integrity and (**B**) global efficiency of functional networks**.** Analyses were conducted separately for healthy controls (HCs), Alzheimer's disease (AD), and frontotemporal lobar degeneration (FTLD) groups. The average gray matter volume intensity and global efficiency of the functional network were predicted using ridge regression with 20 repetitions and 3-fold cross-validation based on Education Quality (EQ) and the interaction of EQ and years of education (EQ x YoE). Adjustments were made for age, sex, and cognition (assessed via the Mini-Mental State Examination). Grey matter integrity and functional connectivity analysis were also adjusted for total intracranial volume (TIV) and resting-state recording conditions (open or closed eyes), respectively. Corrections were applied using the False Discovery Rate (*p*_FDR_ < 0·05). *Abbreviations*: ***: *p* < 0·001, AD: Alzheimer's Disease, EQ: education quality, FTLD: frontotemporal lobar degeneration, HCs: healthy controls, ns: not significant, YoE: years of education.

# S11. Whole-brain analysis adjusted by income and socioeconomic values at country and individual levels

The main effects of EQ and YoE were replicated in the whole-brain analysis after adjusting for income and socioeconomic values at the country level. Country-level Gini index and Gross Domestic Product (GDP) were used for aggregate-level factors included in the whole-brain analysis (Supplementary Figure 5A). We also tested the original results using a subsample of 936 participants, where individual socioeconomic status data was available. In this subsample, we used ESOMAR^19-22^ to obtain an individual metric of socioeconomic status. ESOMAR estimates socioeconomic status based on parental education and occupation, providing a standardized measure for cross-national comparisons.^19-22^ We used the averaged data from both parents for overall participants. ^19-22^ Results are shown in Supplementary Figure 5B.


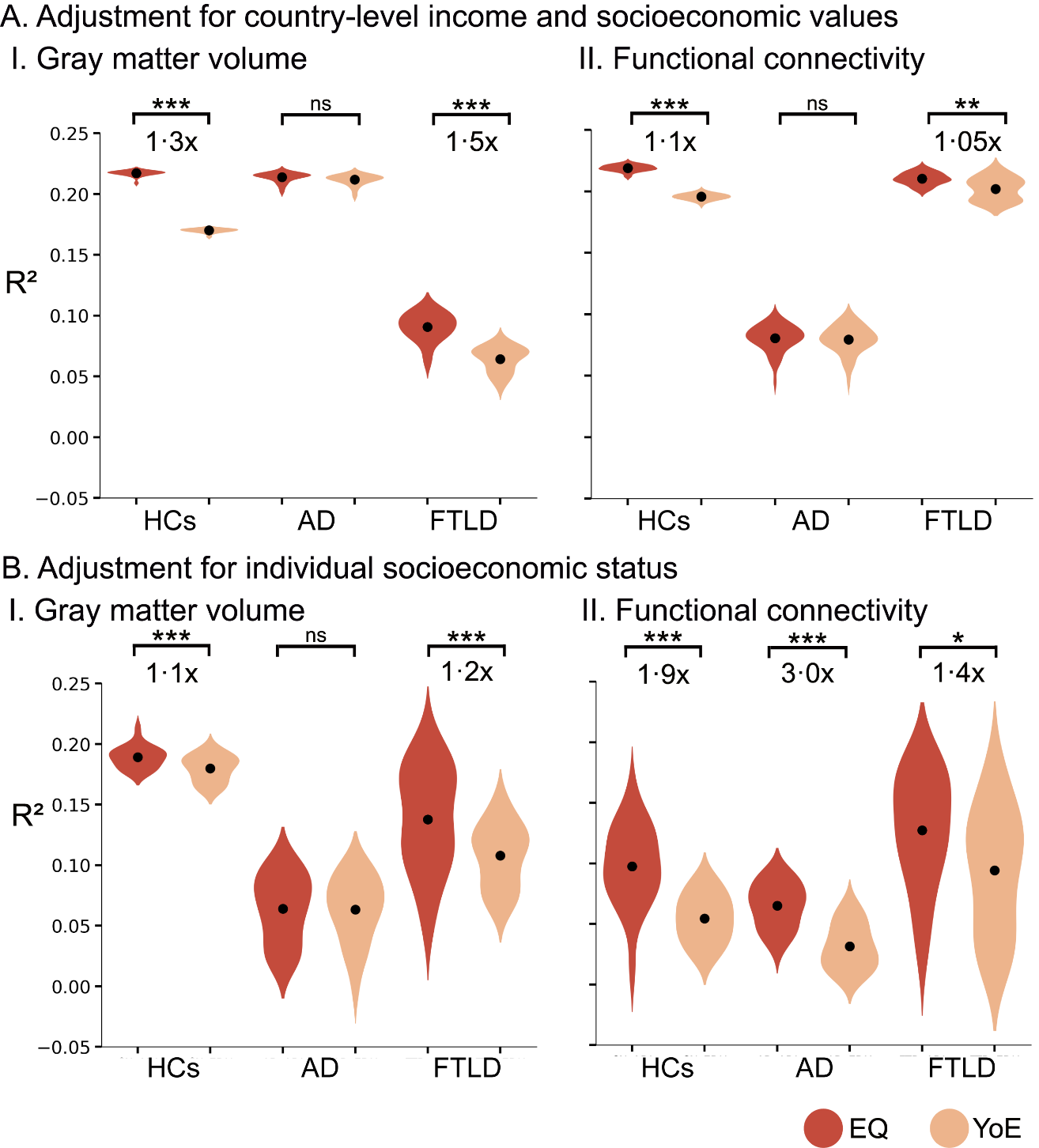


Supplementary Figure 5**.** Impact of education adjusted by income and socioeconomic values at the country (A) and individual (B) levels on whole-brain measures of (**I**) grey matter integrity and (**II**) functional connectivity. Analyses were conducted separately for healthy controls (HCs), Alzheimer's disease (AD), and frontotemporal lobar degeneration (FTLD) groups. The average gray matter volume intensity and global efficiency of the functional network were predicted using ridge regression with 20 repetitions and 3-fold cross-validation based on Education Quality (EQ) and YoE, including the country indexes of Gini and gross domestic product indexes in each analysis. Adjustments were made for age, sex, and cognition (assessed via the Mini-Mental State Examination). Grey matter integrity and functional connectivity analysis were also adjusted for total intracranial volume (TIV) and resting-state recording conditions (open or closed eyes), respectively. We employed the country average Gini index and Gross Domestic Product at the country level across the latest five available reports. At the individual level, we used an individual metric of socioeconomic status. Corrections were applied using the False Discovery Rate (*p*_FDR_ < 0·05). *Abbreviations*: *: *p* < 0·05, **: *p* < 0·01, ***: *p* < 0·001, AD: Alzheimer's Disease, EQ: education quality, FTLD: frontotemporal lobar degeneration, HCs: healthy controls, ns: not significant, YoE: years of education.

# S12. Whole-brain analysis adjusted by differences in sample size across countries


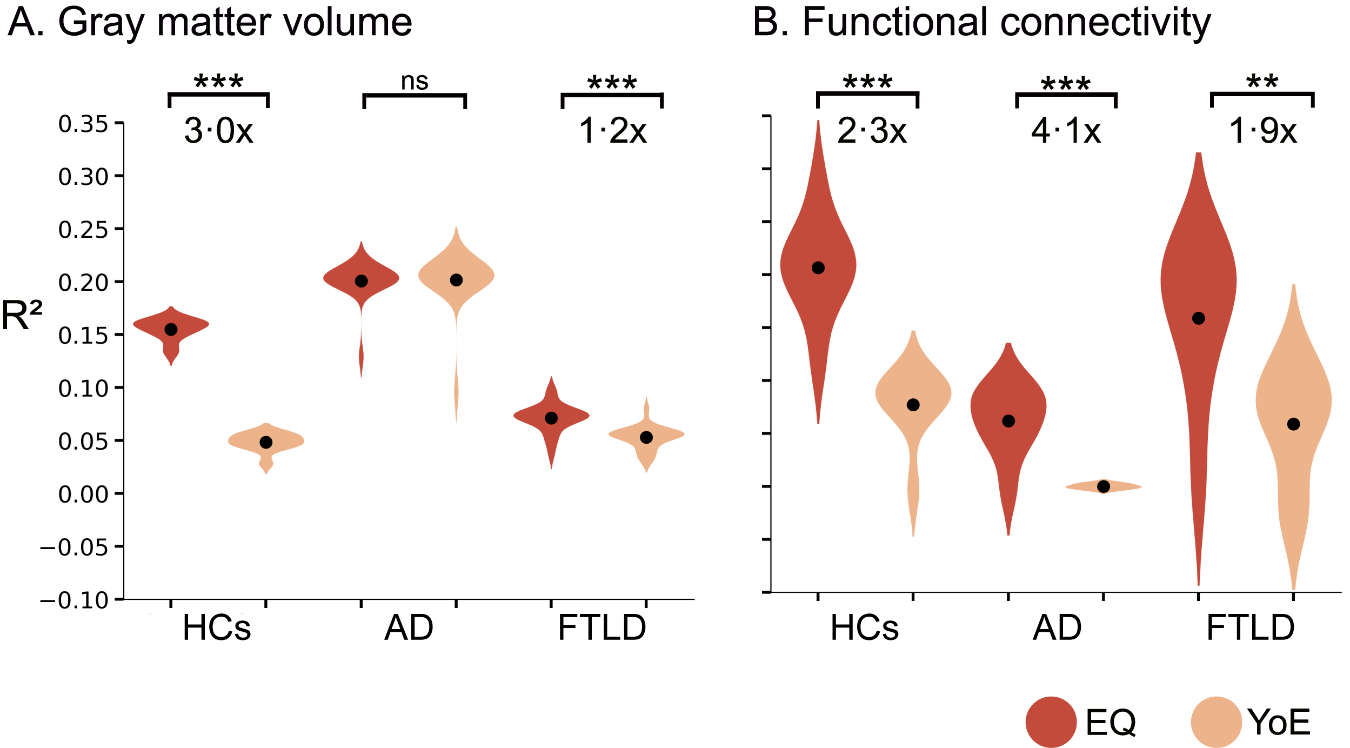
The whole-brain analysis was replicated using a bootstrap approach to discard potential bias driven by different sample sizes across countries.^23^ This analysis replicated the main effects of EQ and YoE (Supplementary Figure 6).

Supplementary Figure 6. Effect of education on whole-brain measures using a bootstrap approach sequentially removing one country's sample at each step on (A) grey matter integrity and (B) functional connectivity. Analyses were conducted separately for healthy controls (HCs), Alzheimer's disease (AD), and frontotemporal lobar degeneration (FTLD) groups. The average gray matter volume intensity and global efficiency of the functional network were predicted using ridge regression with 20 repetitions and 3-fold cross-validation based on Education Quality (EQ) and the interaction of EQ and years of education (EQ x YoE). Adjustments were made for age, sex, and cognition (assessed via the Mini-Mental State Examination). Grey matter integrity and functional connectivity analysis were also adjusted for total intracranial volume (TIV) and resting-state recording conditions (open or closed eyes), respectively. Corrections were applied using the False Discovery Rate (*p*_FDR_ < 0·05). *Abbreviations*: **: *p* < 0·01, ***: *p* < 0·001, AD: Alzheimer's Disease, EQ: education quality, FTLD: frontotemporal lobar degeneration, HCs: healthy controls, ns: not significant, YoE: years of education.

# S13. Whole-brain analysis in groups matched by age


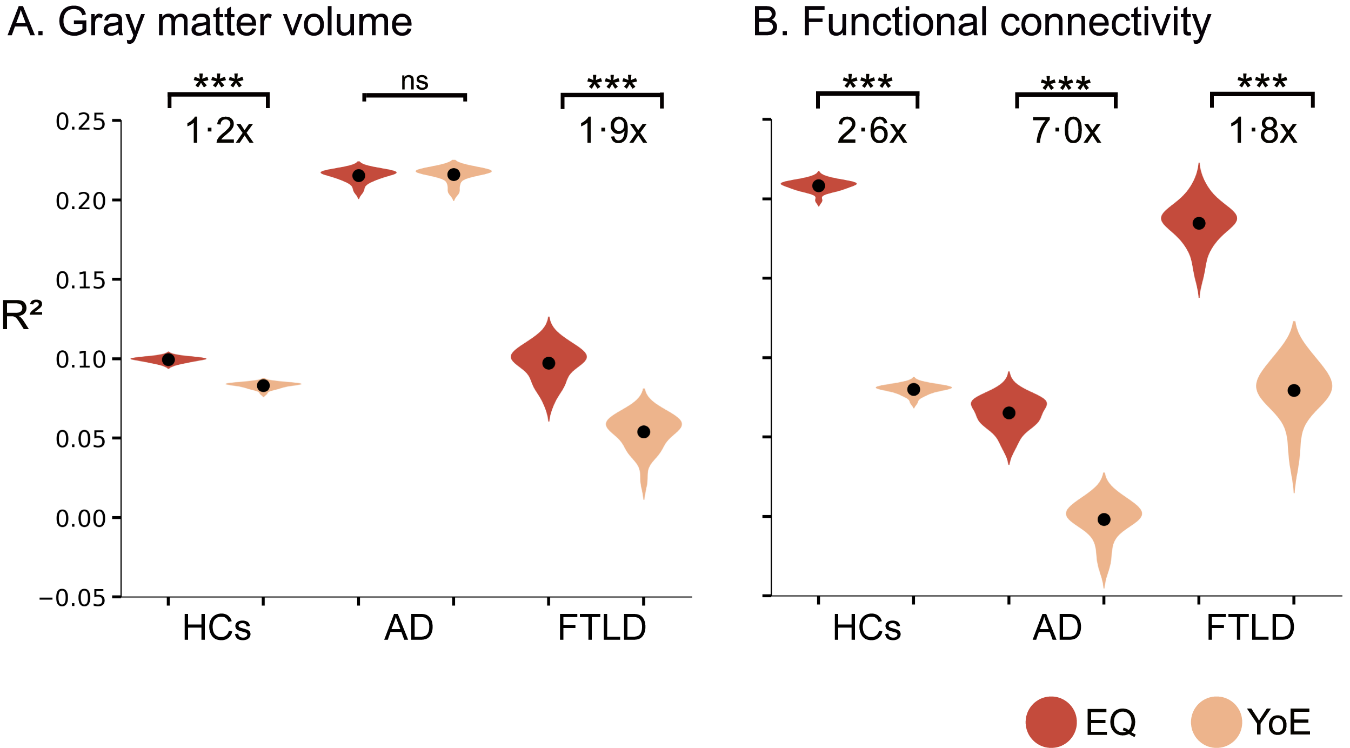
The whole-brain analysis was replicated in age-matched groups. In this subsample, there were no significant age differences in the atrophy (HCs vs AD: *t*=−1·2, *p*=0·23, HCs vs FTLD: *t* = 1·2, *p* = 0·27) or functional connectivity (HCs vs AD: *t*=−3·3,*p*=0·76, HCs vs FTLD: *t* = 1·3, *p* = 0·25) datasets. As a result, the main effects were reproduced. (Supplementary Figure 7), atrophy (HCs: ∆mean= 1·6[1·5–1·7]×10^-2^, *p*<10^-5^; AD: ∆mean= 0·0[-0·03–0·011]×10^-2^, *p*=0·59; FTLD: ∆mean= 4·3[3·7–5·0]×10^-2^, *p*<10^-5^, all with 95% confidence intervals) and functional connectivity (HCs: ∆mean= 12·8[12·7–13·0]×10^-2^, *p*<10^-5^; AD: ∆mean= 6·7[6·1–7·3]×10^-2^, *p*<10^-5^; FTLD: ∆mean= 10·5[9·6–11·5]×10^-2^, *p*<10^-5^, all with 95% confidence intervals).

Supplementary Figure 7. Impact of education on whole-brain measures in a subsample matched by age (**A**) grey matter integrity and (**B**) global efficiency of functional networks**.** Analyses were conducted separately for healthy controls (HCs), Alzheimer's disease (AD), and frontotemporal lobar degeneration (FTLD) groups. The average gray matter volume intensity and global efficiency of the functional network were predicted using ridge regression with 20 repetitions and 3-fold cross-validation based on Education Quality (EQ) and the interaction of EQ and years of education (EQ x YoE). Adjustments were made for age, sex, and cognition (assessed via the Mini-Mental State Examination). Grey matter integrity and functional connectivity analysis were also adjusted for total intracranial volume (TIV) and resting-state recording conditions (open or closed eyes), respectively. Corrections were applied using the False Discovery Rate (*p*_FDR_ < 0·05). *Abbreviations*: ***: *p* < 0·001, AD: Alzheimer's Disease, EQ: education quality, FTLD: frontotemporal lobar degeneration, HCs: healthy controls, ns: not significant, YoE: years of education.

# S14. Whole-brain analysis without adjustment by cognition

The main results were reproduced in whole-brain analysis without controlling for cognition (Supplementary Figure 8).


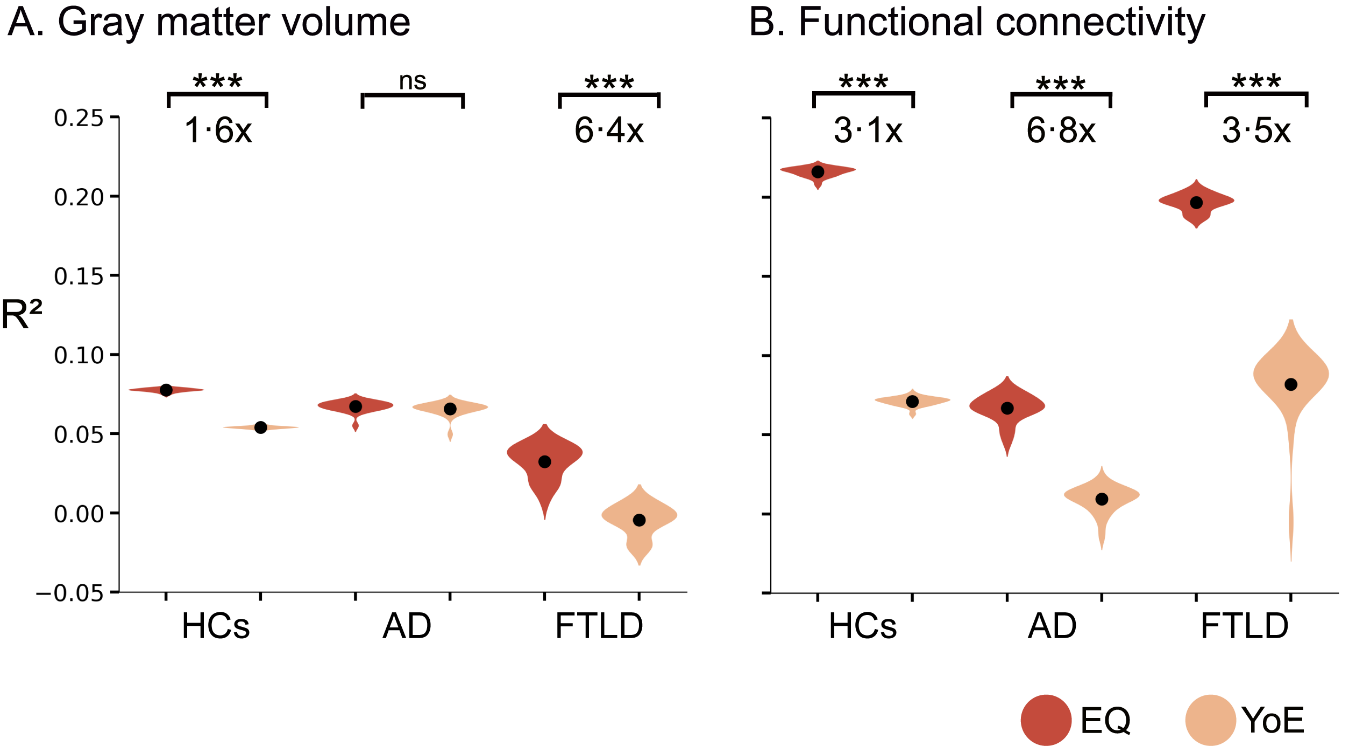


Supplementary Figure 8**.** Impact of education on whole-brain measures of (**A**) grey matter integrity and (**B**) global efficiency of functional networks**.** Analyses were conducted separately for healthy controls (HCs), Alzheimer's disease (AD), and frontotemporal lobar degeneration (FTLD) groups. The average gray matter volume intensity and global efficiency of the functional network were predicted using ridge regression with 20 repetitions and 3-fold cross-validation based on Education Quality (EQ) and the interaction of EQ and years of education (EQ x YoE). Adjustments were made for age and sex. Grey matter integrity and functional connectivity analysis were also adjusted for total intracranial volume (TIV) and resting-state recording conditions (open or closed eyes). Corrections were applied using the False Discovery Rate (*p*_FDR_ < 0·05). *Abbreviations*: ***: *p* < 0·001, AD: Alzheimer's Disease, EQ: education quality, FTLD: frontotemporal lobar degeneration, HCs: healthy controls, ns: not significant, YoE: years of education.

# References

1. Petersen RC, Aisen PS, Beckett LA, et al. Alzheimer's Disease Neuroimaging Initiative (ADNI): Clinical characterization. *Neurology* 2010; **74**: 201-9.

2. Fowler C, Rainey-Smith SR, Bird S, et al. Fifteen Years of the Australian Imaging, Biomarkers and Lifestyle (AIBL) Study: Progress and Observations from 2,359 Older Adults Spanning the Spectrum from Cognitive Normality to Alzheimer's Disease. *J Alzheimers Dis Rep* 2021; **5**(1): 443-68.

3. Frisoni GB. Alzheimer's disease neuroimaging initiative in Europe. *Alzheimers Dement* 2010; **6**(3): 280-5.

4. Shafto MA, Tyler LK, Dixon M, et al. The Cambridge Centre for Ageing and Neuroscience (Cam-CAN) study protocol: a cross-sectional, lifespan, multidisciplinary examination of healthy cognitive ageing. *BMC Neurology* 2014; **14**(1): 204.

5. Nęcka ES, Magdalena; Kucharzyk, Bartłomiej; Falkiewicz, Marcel. Who can afford self-control? The neural efficiency mechanism explains effective self-regulation of behavior. *Stanford Digital Repository* 2015.

6. Nilsonne G, Tamm S, Schwarz J, et al. Intrinsic brain connectivity after partial sleep deprivation in young and older adults: results from the Stockholm Sleepy Brain study. *Sci Rep* 2017; **7**(1): 9422.

7. Aerts H, Colenbier N, Almgren H, et al. Pre- and post-surgery brain tumor multimodal magnetic resonance imaging data optimized for large scale computational modelling. *Sci Data* 2022; **9**(1): 676.

8. Spreng RN, Setton R, Alter U, et al. Neurocognitive aging data release with behavioral, structural and multi-echo functional MRI measures. *Sci Data* 2022; **9**(1): 119.

9. Groessinger D, Fischmeister, F. P. S., Witte, M., Koschutnig, K., Ninaus, M., Neuper, C., Kober, S.E. & Wood, G. The role of superstition of cognitive control during neurofeedback training. *bioRxiv* 2021.

10. Tisdall L, Mugume S, Kellen D, Mata R. Lifespan trajectories of risk preference, impulsivity, and self-control: A dataset containing self-report, informant-report, behavioral, hormone and functional neuroimaging measures from a cross-sectional human sample. *Data Brief* 2024; **52**: 109968.

11. Maekawa T, Sasaoka T, Inui T, Fermin ASR, Yamawaki S. Heart rate and insula activity increase in response to music in individuals with high interoceptive sensitivity. *PLoS One* 2024; **19**(8): e0299091.

12. Rohrer JD, Rosen HJ. Neuroimaging in frontotemporal dementia. *International Review of Psychiatry* 2013; **25**: 221-9.

13. Coronel‐Oliveros C, Gómez RG, Ranasinghe K, et al. Viscous dynamics associated with hypoexcitation and structural disintegration in neurodegeneration via generative whole‐brain modeling. *Alzheimer's & Dementia* 2024; **20**(5): 3228-50.

14. Bookheimer SY, Salat DH, Terpstra M, et al. The Lifespan Human Connectome Project in Aging: An overview. *Neuroimage* 2019; **185**: 335-48.

15. Babayan A, Erbey M, Kumral D, et al. A mind-brain-body dataset of MRI, EEG, cognition, emotion, and peripheral physiology in young and old adults. *Sci Data* 2019; **6**: 180308.

16. Ibanez A, Yokoyama JS, Possin KL, et al. The Multi-Partner Consortium to Expand Dementia Research in Latin America (ReDLat): Driving Multicentric Research and Implementation Science. Frontiers in Neurology: Front Neurol; 2021. p. 631722.

17. Planche V, Manjon JV, Mansencal B, et al. Structural progression of Alzheimer’s disease over decades: the MRI staging scheme. *Brain Communications* 2022; **4**(3).

18. Chu M, Jiang D, Li D, et al. Atrophy network mapping of clinical subtypes and main symptoms in frontotemporal dementia. *Brain* 2024; **147**(9): 3048-58.

19. Hoffmeyer-Zlotnik JHP, Wolf C. The ESOMAR Standard Demographic Classification. Advances in Cross-National Comparison: A European Working Book for Demographic and Socio-Economic Variables. Boston, MA: Springer US; 2003: 97-121.

20. Mtintsilana A, Craig A, Mapanga W, Dlamini SN, Norris SA. Association between socio-economic status and non-communicable disease risk in young adults from Kenya, South Africa, and the United Kingdom. *Sci Rep* 2023; **13**(1): 728.

21. Migeot J, Calivar M, Granchetti H, Ibanez A, Fittipaldi S. Socioeconomic status impacts cognitive and socioemotional processes in healthy ageing. *Sci Rep* 2022; **12**(1): 6048.

22. Martínez-Flórez JF, Belalcázar M, Alvarez A, Erazo O, Sevilla S, Parra MA. Short-term memory binding is insensitive to the socioeconomic status of older adults with and without mild cognitive impairment. *The Clinical Neuropsychologist* 2024; **38**(8): 1947-66.

23. Chi G, Fang H, Chatterjee S, Blumenstock JE. Microestimates of wealth for all low- and middle-income countries. *Proceedings of the National Academy of Sciences* 2022; **119**(3): e2113658119.
